# Improved protocol for efficacious *in vitro* androgenesis and development of doubled haploids in temperate japonica rice

**DOI:** 10.1371/journal.pone.0241292

**Published:** 2020-11-02

**Authors:** Aafreen Sakina, Saba Mir, Sofi Najeeb, Sajad M. Zargar, Firdous A. Nehvi, Zahoor A. Rather, Romesh K. Salgotra, Asif B. Shikari

**Affiliations:** 1 Division of Plant Biotechnology, Sher-e-Kashmir University of Agricultural Sciences and Technology of Kashmir, Srinagar, J&K, India; 2 Mountain Research Centre for Field Crops, Sher-e-Kashmir University of Agricultural Sciences and Technology of Kashmir, Srinagar, J&K, India; 3 Division of Floriculture and Landscaping, Sher-e-Kashmir University of Agricultural Sciences and Technology of Kashmir, Srinagar, J&K, India; 4 School of Biotechnology, Sher-e-Kashmir University of Agricultural Sciences and Technology of Jammu, Srinagar, J&K, India; Lovely Professional University, INDIA

## Abstract

DH (Doubled haploid) is the immortal mapping population and an outcome of single meiotic cycle, contributed from male partner. An improved procedure was developed for high frequency androgenesis in japonica genotypes, K-332 and GS-88 and their F_1_s. A total of 207 fertile, green, di-haploid plants were generated from K-332 × GS-88 hybrids using the improved anther culture protocol. The investigation was carried out to evaluate callus induction potential and regeneration response for the genotypes and the derived F_1_s on N6 media and modified N6 media (N6_M_). Whereas, N6 failed to induce callusing, agarose solidified N6_M_ media supplemented with 4% maltose, growth regulators; NAA (2 mg/l), 2, 4-D (0.5 mg/l), Kinetin (0.5 mg/l), and silver nitrate induced high calli percentage of 27.6% in F_1_s, 9.5% and 6.7% in GS-88 and K-332 respectively. Murashige and Skoog (MS) media supplemented with 3% sucrose, and the hormonal combination BAP (2 mg/l), Kinetin (1 mg/l) and NAA (1 mg/l) induced high green shoot regeneration rates (0–60.0%). The effect of cold pre-treatment at 4°C and the stage of anther collection and their interaction was studied. The effect of cold pre-treatment (CP) of collected boots at 4°C (for CP_2_: 2, CP_4_: 4, CP_6_: 6 and CP_8_: 8 days) at different stages of panicle emergence (BES_4-6_: 4–6, BES_7-10_: 7–10, BES_11-13_: 11–13, BES_>13_: more than 13 inches was worked out in relation to the effect on response of calli induction, albino regeneration, green plant regeneration and number of shoots/green calli. CP referred to the number of days for which the collected boots were incubated before they were inoculated. BES was the length (inches) between flag leaf and penultimate leaf at the time of boot collection. We concluded that CP_6_ and BES_7-10_ showed better response to callus proliferation and regeneration of plantlets across genotypes. The appropriate pre-treatment, stage of anther collection and favourable media composition resulted in high calli induction and green plant regeneration rates in recalcitrant japonica genotypes. The modified N6 media resulted into efficient callus induction and is expected to be useful for studies which aim at rapid generation of mapping populations for genetic studies.

## Introduction

Doubled haploids (DHs) are plants derived from a single isolated microspore which undergoes diploidization either naturally or artificially to form homozygous diploids. Guha and Maheshwari [1] pioneered in reporting on discovery of androgenic haploidy that ensued number of studies on androgenic development route in number of crops. The first successful report on the production of haploid plants in japonica rice was put forward by Niizeki and Oono [2]. Androgenesis holds great promise for generating a myriad of mapping populations for genetic and genomic studies that cover gene and QTL mapping for traits of economic importance [3]. Development of a reliable and efficient regeneration system, including callus induction and differentiation or plant regeneration is pre-requisite for successful development of doubled haploid mapping population in rice. The improvement of the androgenic process can be brought about through better understanding the mechanisms that induce the development pathway involved in switching from gametophytic to sporophytic development. Anther culture technique entails a two-step process: a: calli induction from microspores present within the anther sacs, b: regeneration of green plants from the induced calli. Successful androgenesis depends upon a number of factors such as genotype of pollen donor, stage of pollen development, panicle pre-treatment, culture medium, plant growth regulators, type of sugar and addition of adjuvant, etc. [4]. In general, japonica rice exhibits good androgenic response in N6 media. The modifications in the source and level of nitrogen and carbon, change in concentration of other micronutrients or addition of undefined substances are known to cause varying efficacy of anther culture response [5]. Additionally, the study on the effect of culture media on the anther culture responsiveness in relation to genotype interaction requires a major attention. Since, the anthers bearing microspores at the mid-uninucleate stage show high callus induction [6], we assumed that the particular stage can be timed as per the appropriate stage of boot collection and therefore, was worked out in the present experiment. In case of rice, the most preferable shock pre-treatment delivered to improve the androgenesis is cold pre-treatment of harvested boots. The boots have been subjected to a range of temperatures varying from 4 to 12°C administered for 7–30 days. Even the process of standardization for each genotype has also been advocated [7]. In our study, we have reported the optimization of culture media and regeneration protocol for high frequency androgenesis and green plant regeneration from anthers of two genotypes, K-332 and GS-88 and the resulting F_1_s. The study highlights the ideal stage of pollen harvesting and pre-treatment process that may be vital for improvement of efficiency of anodrogenesis response in rice.

## Materials and methods

### Experimental materials

K-332 is a short grained temperate japonica rice variety grown in high altitude (>2000 msl) regions of Kashmir valley [8]. GS-88 is a bold grained genotype carrying resistance to few biotic stresses like Bakanae disease [9]. The anther culture response of japonica rice genotypes namely, K-332, GS-88 and the derived F_1_ was studied. For validation of standard N6 media Chu [10], widely used for anther culture in rice, we selected few more (japonica) accessions like, GSL-19, IRBL-Pita^2^ and IRBL-Pita and a popular Basmati variety, Pusa Basmati 1509. These four additional lines were included at the start of experiment to support our observations on K-332 and GS-88, should there have been any failure due to media x genotype interaction. The experiment was carried out at Mountain Research Centre for Field Crops, Khudwani, and Division of Plant Biotechnology, Sher-e-Kashmir University of Agricultural Sciences and Technology of Kashmir during the years 2017 to 2019.

### Standardization of rice androgenesis on N6 and N6_M_ media

#### Media preparation

Presently, two different media namely, N6 media and a modified N6 media (N6_M_) media were employed for *invitro* pollen culture of the six genotypes. Media were supplemented with different combinations and concentrations of 2, 4-D, Kinetin and NAA. Both the media were supplemented uniformly with sugar (maltose 40g/L) and an ethylene inhibitor (silver nitrate 5mg/L). In addition, N6 media was supplemented with NAA (2 mg/l), 2, 4-D (0.5 mg/l), Kinetin (0.5 mg/l), cysteine (40 mg/l) and tryptophan (25 mg/l). N6_M_ media was supplemented with NAA (2 mg/l), 2, 4-D (0.5 mg/l) and Kinetin (0.5 mg/l), maltose 4%, silver nitrate 5 mg/L solidified with 5% agarose.

#### Shoot regeneration media and conditions

Murashige and Skoog (MS) media supplemented with different concentrations of BAP (1–3 mg/L), Kinetin (0.5–1 mg/L) and NAA (0.5–1 mg/L) was used for shoot regeneration. Androgenic calli of 2–3 mm diameter were transferred to 100 ml test tubes/ flasks containing regeneration media (S1 Fig). The calli were aseptically transferred under UV sterilized laminar hood. The plated calli were incubated with a 16-h light/ 8-h dark regime at temperature (25±5°C) and 80% RH under artificial light (2000 lux).

### Calli induction and regeneration response

#### Validation of crosses for hybridity

Prior to *invitro* pollen culture, all the putative F_1_s derived from the cross between K-332 × GS-88 were tested for hybridity using SSR markers. Genomic DNA of parents and F_1_s was extracted from young leaves through cetyltrimethyl ammonium bromide (CTAB) method [11] with certain modifications. Parental polymorphism survey was carried out between the two parents using 35 SSR markers through standard PCR assay. Amplification reactions were performed in volumes of 10 μL containing 1x PCR buffer (10 mM Tris, pH 8.4, 50 mM KCl, 1.8 mM MgCl2), 2mM dNTPs (MBI, Fermentas, Lithuania, USA), 5 pmol each of forward and reverse primer and 5U of Taq DNA polymerase (MBI, Fermentas, Lithuania, USA) and 25ng of template DNA. *In vitro* amplification using polymerase chain reaction (PCR) was performed in a 96 well plate in an Eppendorf Machine (Eppendorf, Hamburg, Germany) with following thermal regimes, Ist cycle of 5 min at 94°C followed by 35 cycles of each of 1 min denaturation at 94°C, 1 min annealing at 50–55°C and 2 min extension at 72°C and final extension of 7 min at 72°C. The PCR products were resolved on 3% Electrophoresis Matrix low EEO agarose (G Biosciences, St. Louis, MO, USA) gel stained with 0.5 μg/ml ethidium bromide and visualized in a Gel Documentation System (BioRad, USA). 2 μl of 100 bp DNA ladder (Fermentas, USA) was used to estimate PCR fragment size. Of 35 SSR markers, four were found to be polymorphic and were used for screening of F_1_ plants for hybridity. Anthers from confirmed hybrids were used for pollen culture.

#### Anther pre-treatment and inoculation

Spikelets were harvested from healthy boots at four different stages of development inside leaf sheath. Boot emergence stage (BES) determined and recorded as the distance between flag leaf and penultimate leaf was observed as length measures of 4–6 (BES_4-6_), 7–10 (BES_7-10_), 11–13 (BES_11-13_) and >13 inches (BES_>13_) (S2 Fig). The boots were collected during morning hours (9 to 10 am) from primary plant tillers with intact penultimate leaf sheath and were wiped 2–3 times using a clean muslin cloth moistened with 70% ethanol. Subsequently, the boots were wrapped in clean aluminum foil and enclosed in polythene bags in order to prevent desiccation. The wrapped boots were incubated at 4°C for 2, 4, 6 and 8 days after first incubation. At the time of anther inoculation, the cold pre-treated spikelets removed from the leaf sheath were trimmed to retain up to middle portion of the panicle. Spikelets were collected in a beaker and immersed in Bavistin solution (1g/L) with few drops of Tween-20 for 20 mins with occasional shaking. Spikelets were washed with running water and finally rinsed three times in sterile distilled water. The treated spikelets were again sterilized with 0.1% mercuric chloride solution for 7 minutes under asceptic conditions in laminar air hood. The mercuric chloride was drained out and florets were rinsed three times with autoclaved double distilled water to remove traces of adhered mercuric chloride. Anthers were dissected out from the florets by holding them from their tips with a sterile forcep and snipping florets at their base, with the help of a sterile scissor to detach the anther lobes from the filaments. The cut floret was tapped on the rim of the culture tube so that anthers drop down onto the media.

#### Calli induction

Inoculation was done on N6_M_ media supplemented with NAA (2 mg/l), 2, 4-D (0.5 mg/l) and Kinetin (0.5 mg/l), maltose 4%, silver nitrate (5 mg/l) solidified with 5% agarose. The anther inoculated test tubes were checked occasionally to record observations on callus induction and to remove infected tubes.

#### Shoot regeneration media

Murashige and Skoog (MS) media supplemented with 3% sucrose, BAP (2 mg/L), Kinetin (1 mg/L) and NAA (1 mg/L) and solidified with 8% agar was used for shoot regeneration.

#### Root regeneration

Green shoots having 3–4 inches length were transferred to rooting media for vigorous root development. Rooting media comprised of MS media supplemented with 3% sucrose and solidified with 8% agar at a pH of 5.8. No hormones were added to the media. Media was dispensed in 500 ml flasks to ensure sufficient space for healthy growth of seedlings.

#### Hardening and acclimatization

The regenerates with well-developed roots were removed from media with the help of scalpels and the residual agar attached to roots was thoroughly washed off in tap water. The plantlets were transferred to plastic cups containing hardening media (autoclaved pre-soaked vermiculite & perlite in the ratio of 1:1) inside the growth chamber. The cup was covered with another cup to maintain humidity for few days and thereafter removed. 80% RH and 28°C temperature was maintained inside the growth chamber. After hardening for 15 days in growth chamber, plants were transferred to the soil in the mud pots and were kept in green house and maintained at 30°C with 70% relative humidity for the period of 30 days and later transferred to field in open air conditions.

*Evaluation of DHs for agronomic traits*. The first generation pollen derived plants were grown in pots and evaluated for grain type and spikelet fertility. The progenies obtained from DHs were sown in irrigated field conditions along 3m rows at a spacing of 20x15 inches. The observations on five random plants was recorded for the traits, plant height, panicle length, effective tillers per plant, spikelets per panicle and grain yield per plant.

#### Statistical analysis

The anthers from two genotypes, K-332 and GS-88 and the derived F_1_s were inoculated in test tubes with 50 anthers per tube. The experiment was laid in Factorial Complete Randomized Design [12]. Observations were recorded on callus induction, green plant regeneration, albino regeneration, total regeneration and number of shoots/green calli at appropriate stage. The Callus induction frequency (CIF) was obtained from the number of calli against the total number of explants inoculated. Similarly, the Green plantlet differentiation frequency (GPDF) was calculated as the number of green plantlets regenerated as percentage of the number of transferred calli. The albino differentiation frequency was calculated on the number of albino plants and expressed as percentage of the number of transferred calli. Total regeneration was calculated on the number of both albino and green plantlet differentiation as percentage of the number of transferred calli. Number of shoots was calculated on the number of regenerated shoots as percentage of the number of transferred green calli. Callus induction ability was measured as the number of calli induced per 100 anthers inoculated. The data were subjected to log transformation for performing ANOVA using Windostat 9.3 software. Partitioning of means squares were carried out for parental and F_1_ main effects using Group Balanced Block Design as per Gomez & Gomez [12]. A single degree of freedom interaction component for parents vs F_1_s was worked out as per the modification by Shikari and Sinhamahapatra [13].

## Results

### Validation of crosses for hybridity

**Fig 1:**
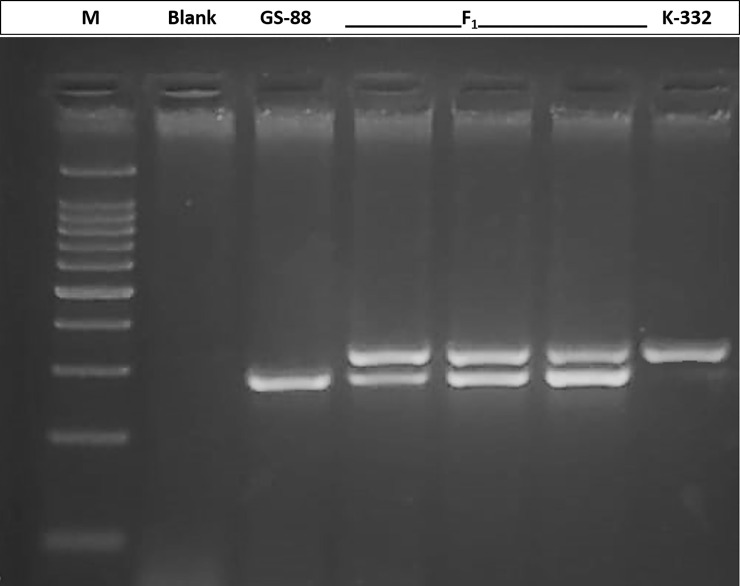
Confirmation of hybridity of F_1_ individuals with polymorphic SSR marker RM72. M: 100 bp DNA ladder (Genetix Biotech Asia, New Delhi, India), C: Blank.

A set of 35 SSR markers were assayed for establishment of parental polymorphism between the parents K-332 and GS-88, of which four (11.4%) markers were found to be polymorphic (S3 Fig). Only two polymorphic markers namely, RM72 and RM1137 were used in confirmation of hybridity across 32 putative F_1_ individuals (Fig 1). The F_1_ plants confirmed for heterozygosity at the corresponding loci were finally selected for pollen culture and subsequent development of Doubled Haploid population.

### Standardization of culture media

#### Calli induction

A preliminary exercise was done to evaluate the calli induction potential in six rice genotypes namely, GS-88, K-332, GSL-19, Pusa Basmati 1509, IRBLPita, IRBLPita^2^ and derived F_1_s on N6 media. It was observed that the inoculated anthers turned light brown in color and swelled with complete loss of calli induction. The callus induction in anthers was followed till three months post inoculation but none of the genotypes responded. Culture media is reported to have a considerable impact on induction of the calli from anthers, while some generalization about the media components can be made, the requirements vary from one genotype to another. Therefore, a modified N6 media (N6_M_) was formulated by overall change in the concentration of various macro and micro nutrients_._ The concentration of NO_3_ (3250 mg/l) and NH_4_ (300 mg/l) ions of media was reduced in N6_M_ media compared to N6 media where concentration of NO_3_ ions is 3535 mg/l and NH_4_ ions is 463 mg/l ions. However, the ratio of NO_3:_ NH_4_ was increased. In addition, concentration of all mesos components which are very critical for tissue culture was also increased. The concentration of CaCl_2_ was increased from 166 mg/l in N6 to 440 in N6_M_ media, similarly concentration of MgSO_4_ in N6_M_ media is 360 mg/l as against 185 mg/l in N6 and concentration of KH_2_PO_4_ is 550 mg/l in N6_M_ and 400mg/l in N6. Following the previous research which demonstrated the positive effect of CuSO_4_ on anther culture in barley, CuSO_4_ (0.025 mg/l) was also included in the N6_M_ media [14] (Table 1), besides the use of hormones, NAA, 2-4-D and Kinetin. After 20 days of incubation at 25±2°C in total darkness, anthers first turned light brown and then swelled up followed by bursting out of callus from the middle of anthers. The callusing frequency noted was 2.2% with compact and light yellow calli.

**Table 1:** Comparison of macro and micro nutrient concentrations in N6 and N6_M_ media.

Components (mg/L)	Media	
	N6	N6_M_
(NH4)_2_SO4	463	300
KNO3	3535	3250
KH2PO4	400	550
MgSO4.7H2O	185	360
CaCl2.2H2O	166	440
H3BO3	22.3	6.54
MnSO4.4H2O	8.6	22.3
ZnSO4.7H2O	0.25	8.6
Na2MoO4.2H2O	0.8	0.25
KI	0.025	0.8
CuSO4.5H2O	-	0.025
CoCl2.6H2O	0.025	0.025
FeSO4.7H2O	27.8	27.7
Na2EDTA	37.5	37.3
Thiamine-HCl	2.5	2.5
Nicotinic acid	2.5	2.5
Pyridoxine-HCl	2.5	2.5
Glycine	2	2.5
Inositol	100	100

#### Shoot induction

After 15 days of culture, calli started differentiating into nodular structure and turned into green colour, which subsequently formed shoots. Calli showed differential response in the regeneration media (MS) supplemented on varying hormonal combinations. Calli incubated in MS media supplemented with Combination A [BAP (1 mg/l), Kinetin (1 mg/l) and NAA (0.5 mg/l)] did not regenerate into shoots. Calli incubated in MS media supplemented with Combination B [BAP (1.5 mg/l), Kinetin (0.5 mg/l) and NAA (0.5 mg/l)] regenerated roots and no shoots. Only shoots developed from calli incubated on MS media supplemented using Combination C [BAP (3 mg/l), Kinetin (0.5 mg/l) and NAA (0.5 mg/l)]. Media with Combination D [BAP (2 mg/l), Kinetin (1 mg/l) and NAA (1 mg/l)] developed both roots and shoots and was subsequently used for regeneration in development of DH individuals.

#### Rooting and acclimatization

Plantlets were transferred to hormone free MS supplemented with 3% sucrose for profuse growth of shoots and roots. Well-developed seedlings with profuse roots were subjected to hardening under greenhouse conditions and recorded 100% survival.

### The effect of Cold incubation Period (CP) and stage of boot emergence (BES) on androgenesis in N6_M_ media

#### Calli induction

**Fig 2:**
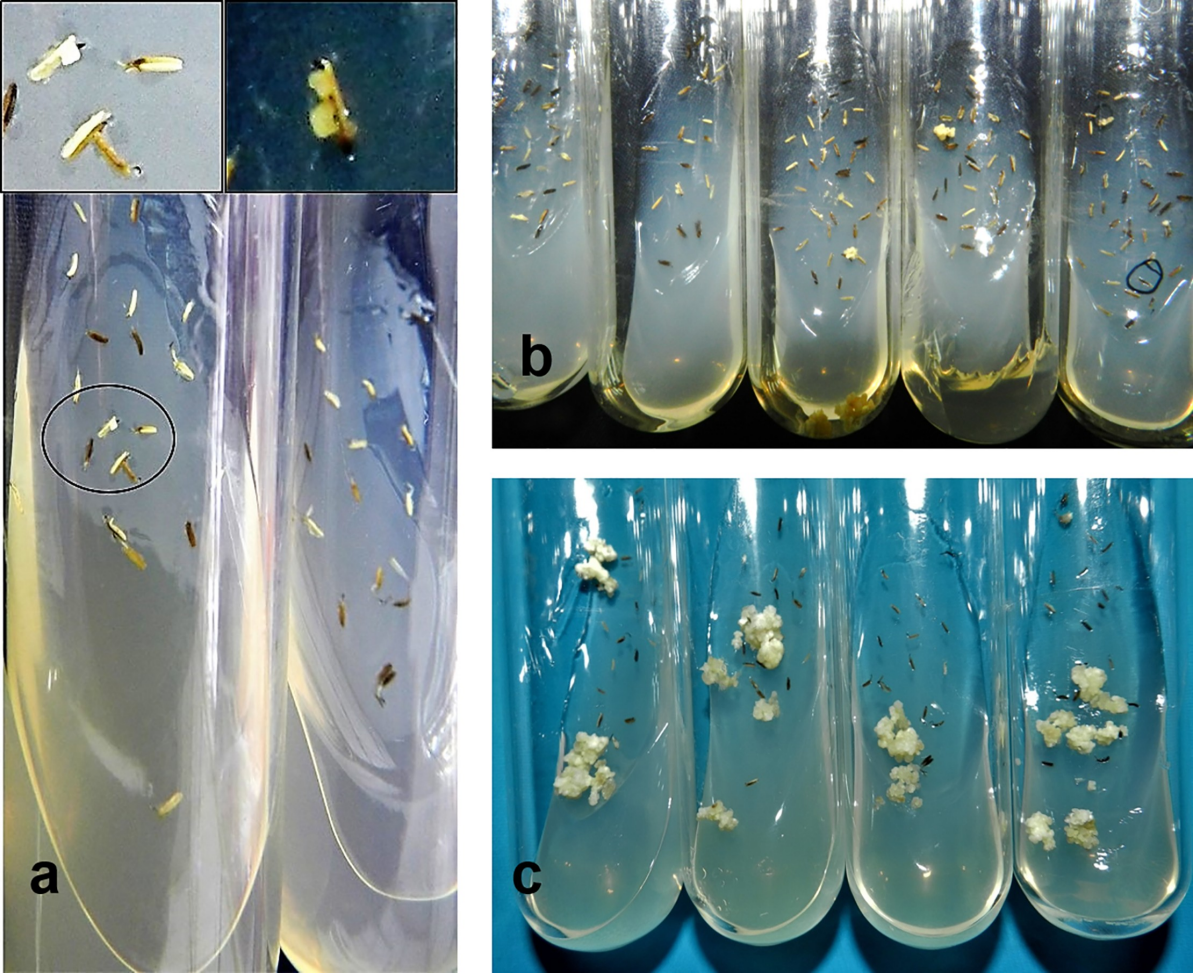
Calli induction and proliferation of F_1_ derived pollen. a: Callus initiation, b: Initial growth, c: Proliferation of white nodular calli from anthers inoculated on N6_M_ media.

Calli induction commenced 15 days after anther inoculation on AA media at all the treatment levels. Prior to calli formation, anthers looked swollen, turned necrotic and calli were seen teeming out from anthers along the middle ridge. Development of multiple calli from a single anther was observed having creamy white and nodular appearance (Fig 2). ANOVA revealed significant mean squares with respect to effect of CP (P<0.001), BES (P<0.001) and CP x BES (P<0.05) on calli induction response, calculated individually for two parents and the derived F_1_. Among four CP levels, CP_6_ promoted maximum calli induction followed by CP_4_ while the treatments CP_2_ and CP_8_ were least effective and showed non-significant difference among themselves. Main effect of BES was significant at P<0.05 with BES_7-10_ better than BES_11-13_ followed equally by BES_4-6_ and BES_>13_. Out of the 16 factorial combinations, the effect of CP for 6 days (CP_6_) and BES of 7–10 inches (BES_7-10_) was found to be uniformly efficient across parents and F_1_. Calli induction frequency varied from 0 (CP_2_BES_>13_) to 27.6% (CP_6_BES_7-10_) in F_1_ derived pollen. In GS-88, calli induction frequency ranged from 0 (CP_2_BES_>13_) to 9.5% (CP_6_BES_7-10_). Following the same pattern, values between 0 (CP_2_BES_>13_) to 6.7% (CP_6_BES_7-10_) were observed for K-332 (Tables 2 and 3, S1 File).

**Table 2:** Mean sum of squares for androgenesis response in relation to Cold Pre-treatment (CP) and Boot Emergence Stage (BES).

Source of Variation	d.f.	Calli induction			Albino regeneration			Green plant regeneration			Total regeneration			Number of shoots/ green calli		
		K-332 x GS-88	GS-88	K-332	K-332 x GS-88	GS-88	K-332	K-332 x GS-88	GS-88	K-332	K-332 x GS-88	GS-88	K-332	K-332 x GS-88	GS-88	K-332
CP	3	2.99**	1.54**	1.26**	2.45**	1.99**	2.17**	4.26**	2.35**	2.62**	5.40**	2.99**	5.22**	8.53**	6.69**	7.07**
BES	3	3.97**	2.35**	1.75**	5.54**	5.00**	3.37**	6.27**	5.29**	5.33**	15.01**	13.37**	10.88**	13.59**	14.54**	11.81**
CP x BES	9	0.24*	0.10*	0.12**	0.80	0.56	0.45	0.81*	0.60	0.85**	0.84	0.47	0.91*	2.17**	2.79**	2.07**
Error	160	0.11	0.05	0.12**	0.43	0.39	0.37	0.40	0.32	0.27	0.54	0.5	0.42	0.77	0.79	0.62

CP: Cold pre-treatment for CP_2_: 2, CP_4_: 4, CP_6_: 6 and CP_8_: 8 days at 4°C; BES: Boot emergence stage of BES_4-6_: 4–6, BES_7-10_: 7–10, BES_11-13_: 11–13 and BES_>13_: >13 inches.

**Table 3:** Main and interaction effects pronounced by Cold Pre-treatment (CP) and Boot Emergence Stage (BES) on callus induction and number of shoots per green callus.

Genotype	Parameter	Treatment	BES_4-6_	BES_7-10_	BES_11-13_	BES_>13_	Mean±S.Em.	C.D. (P<0.05)	F Prob.
**F**_**1**_	**Callus Induction**	**CP**_**2**_	0.09 (0.5)^Bb^	0.46 (2.7)^Aa^	0.17 (0.9)^Bb^	0.02 (0.0)^Bb^	0.18 a±0.07	0.22	0.001
		**CP**_**4**_	0.09 (0.6)^Cc^	0.97 (10.5)^Aa^	0.76 (6.6)^Aa^	0.41 (2.5)^Bb^	0.56 b±0.10	0.30	0.000
		**CP**_**6**_	0.35 (2.7)^Cc^	1.36 (27.6)^Aa^	0.71 (6.0)^Bb^	0.57 (3.9)^Bbc^	0.75 c±0.11	0.31	0.000
		**CP**_**8**_	0.08 (0.4)^Bb^	0.56 (5.5)^Aa^	0.34 (2.6)^Aab^	0.09 (0.6)^Bb^	0.27 a±0.11	0.32	0.012
		**Mean**	0.15^A^	0.84^B^	0.49^C^	0.27^A^	0.44±0.05	0.14	0.000
	**Number of shoots**	**CP**_**2**_	0.01 (0.0)^Cc^	2.28 (190.9)^Aa^	1.66 (45.5)^Bb^	0.01 (0.0)^Cc^	0.99 a±0.24	0.68	0.018
		**CP**_**4**_	0.01 (0.0)^Bb^	2.47 (297.0)^Aa^	2.62 (418.2)^Aa^	2.10 (127.3)^Aa^	1.80 b±0.35	1.01	0.006
		**CP**_**6**_	0.01 (0.0)^Cc^	2.73 (531.8)^Aa^	1.96 (90.9)^Aa^	1.80 (63.6)^Bab^	1.62 b±0.29	0.82	0.000
		**CP**_**8**_	0.01 (0.0)^A^	1.66 (45.5)^A^	0.01 (0.0)^A^	0.01 (0.0) ^A^	0.42 c±0.12	_	0.403
		**Mean**	0.01 ^A^	2.28^B^	1.56^C^	0.98^D^	1.20±0.13	0.37	0.000
**K-332**	**Callus Induction**	**CP**_**2**_	0.01 (0.0)^Bb^	0.22 (1.1)^Aa^	0.09 (0.4)^Bab^	0.01 (0.0)^Bb^	0.08 a±0.05	0.15	0.025
		**CP**_**4**_	0.13 (0.6)^Cc^	0.67 (4.4)^Aa^	0.39 (1.6)^Bb^	0.09 (0.4)^Cc^	0.32 b±0.06	0.19	0.000
		**CP**_**6**_	0.18 (0.7)^Cc^	0.87 (6.7)^Aa^	0.48 (2.5)^Bb^	0.22 (1.1)^Cc^	0.45c±0.06	0.19	0.000
		**CP**_**8**_	0.09 (0.4)^Bb^	0.39 (1.6)^Aa^	0.01 (0.0)^Bab^	0.01 (0.0)^Bb^	0.14 a±0.06	0.18	0.013
		**Mean**	0.10^A^	0.52^B^	0.27^C^	0.09^A^	0.25±0.03	0.08	0.000
	**Number of shoots**	**CP**_**2**_	0.01 (0.0)^B^	1.91 (81.8)^A^	0.01 (0.0)^B^	0.01 (0.0)^B^	0.48 A±0.16	_	0.101
		**CP**_**4**_	0.01 (0.0)^Cb^	2.45 (281.8)^Aa^	2.19 (154.5)^Bb^	0.01 (0.0)^Cb^	1.16 B±0.27	0.79	0.000
		**CP**_**6**_	0.01 (0.0)^Bb^	2.71 (513.6)^Aa^	1.80 (63.7)^Bb^	0.01 (0.0)^Bb^	1.13 B±0.33	0.94	0.000
		**CP**_**8**_	0.01 (0.0)^A^	1.66 (45.5)^A^	0.01 (0.0)^A^	0.01 (0.0)^A^	0.42 A±0.12	_	0.403
		**Mean**	0.01^C^	2.18^A^	1.04^B^	0.01^BC^	0.42±0.11	0.33	0.000
**GS-88**	**Callus Induction**	**CP**_**2**_	0.01 (0.0)^Cb^	0.34 (1.6)^Aa^	0.13 (0.6)^Bb^	0.01 (0.0)^Cb^	0.12 a±0.05	0.15	0.000
		**CP**_**4**_	0.18 (0.7)^Cc^	0.74 (5.5)^Aa^	0.45 (2.2)^Bb^	0.13 (0.6)^Cc^	0.37 b±0.07	0.21	0.000
		**CP**_**6**_	0.24 (1.1)^Cc^	1.00 (9.5)^Aa^	0.54 (3.1)^Bb^	0.37 (1.6)^Cbc^	0.54 c±0.07	0.20	0.000
		**CP**_**8**_	0.13 (0.6)^Bbc^	0.46 (2.6)^Aa^	0.22 (0.9)^Bab^	0.01 (0.0)^Cc^	0.19 a±0.07	0.21	0.005
		**Mean**	0.14^A^	0.62^B^	0.34^C^	0.13^A^	0.31±0.03	0.09	0.000
	**Number of shoots**	**CP**_**2**_	0.01 (0.0)^Cc^	2.45 (90.9)^Aa^	1.26 (18.2)^Bb^	0.01 (0.0)^Cc^	0.93 a±0.26	0.75	0.016
		**CP**_**4**_	0.01 (0.0)^Cc^	2.27 (186.4)^Aa^	2.30 (236)^Aa^	1.44 (27.3)^Bb^	1.50 b±0.36	1.04	0.001
		**CP**_**6**_	0.01 (0.0)^Cc^	2.58 (381.8)^Aa^	1.86 (72.7)^Aab^	1.66 (45.5)^Bb^	1.52 b±0.26	0.76	0.000
		**CP**_**8**_	0.01 (0.0)^A^	1.66 (45.5)^A^	0.01 (0.0)^A^	0.01 (0.0)^A^	0.42 c±0.12	_	0.403
		**Mean**	0.01 ^A^	2.24 ^B^	1.35 ^C^	0.78 ^D^	1.09±0.13	0.37	0.000

CP: Cold pre-treatment for CP_2_: 2, CP_4_: 4, CP_6_: 6 and CP_8_: 8 days at 4°C; BES: Boot emergence stage of BES_4-6_: 4–6, BES_7-10_: 7–10, BES_11-13_: 11–13 and BES_>13_: >13 inches; Values given are Log_10_ transformed; Figures in parenthesis denote actual percentages.

#### Shoot regeneration

**Fig 3:**
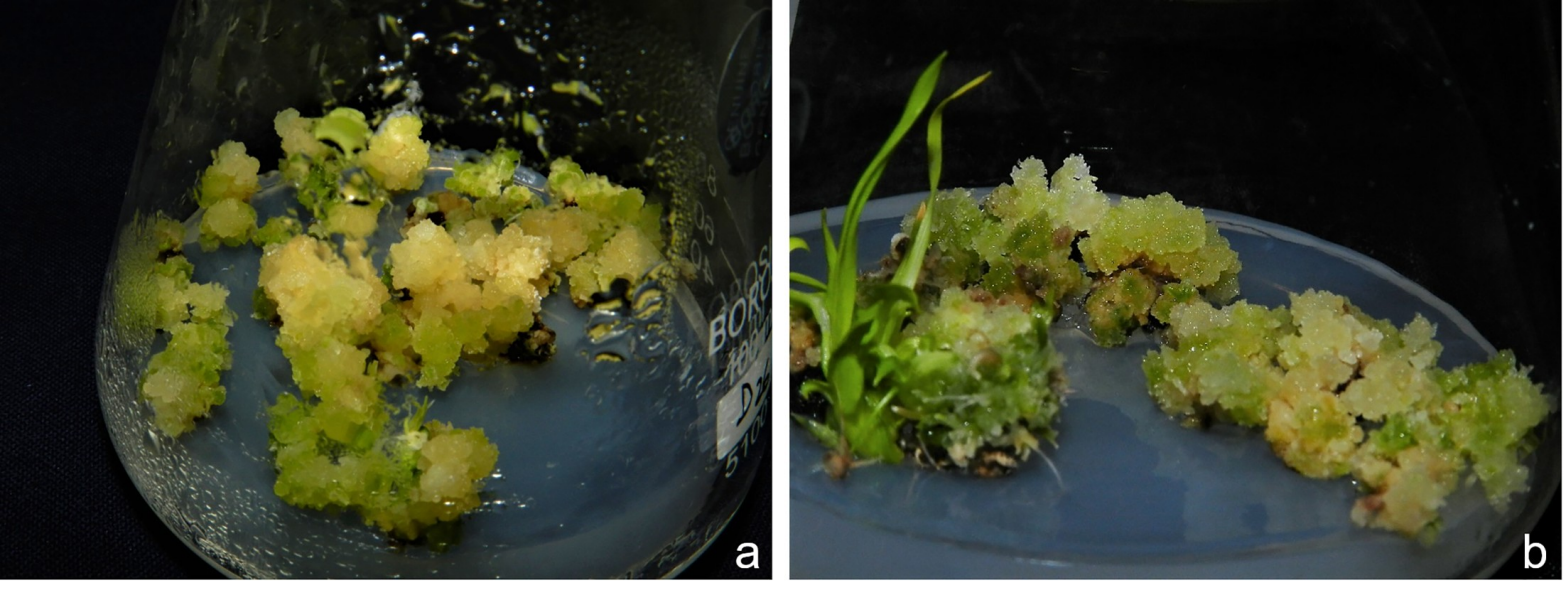
Green plant regeneration from F_1_ derived pollen on N6_M_ media. a: Emergence of green spots in the pollen derived calli inoculated on regeneration media b. Development of shoots from green calli.

**Fig 4:**
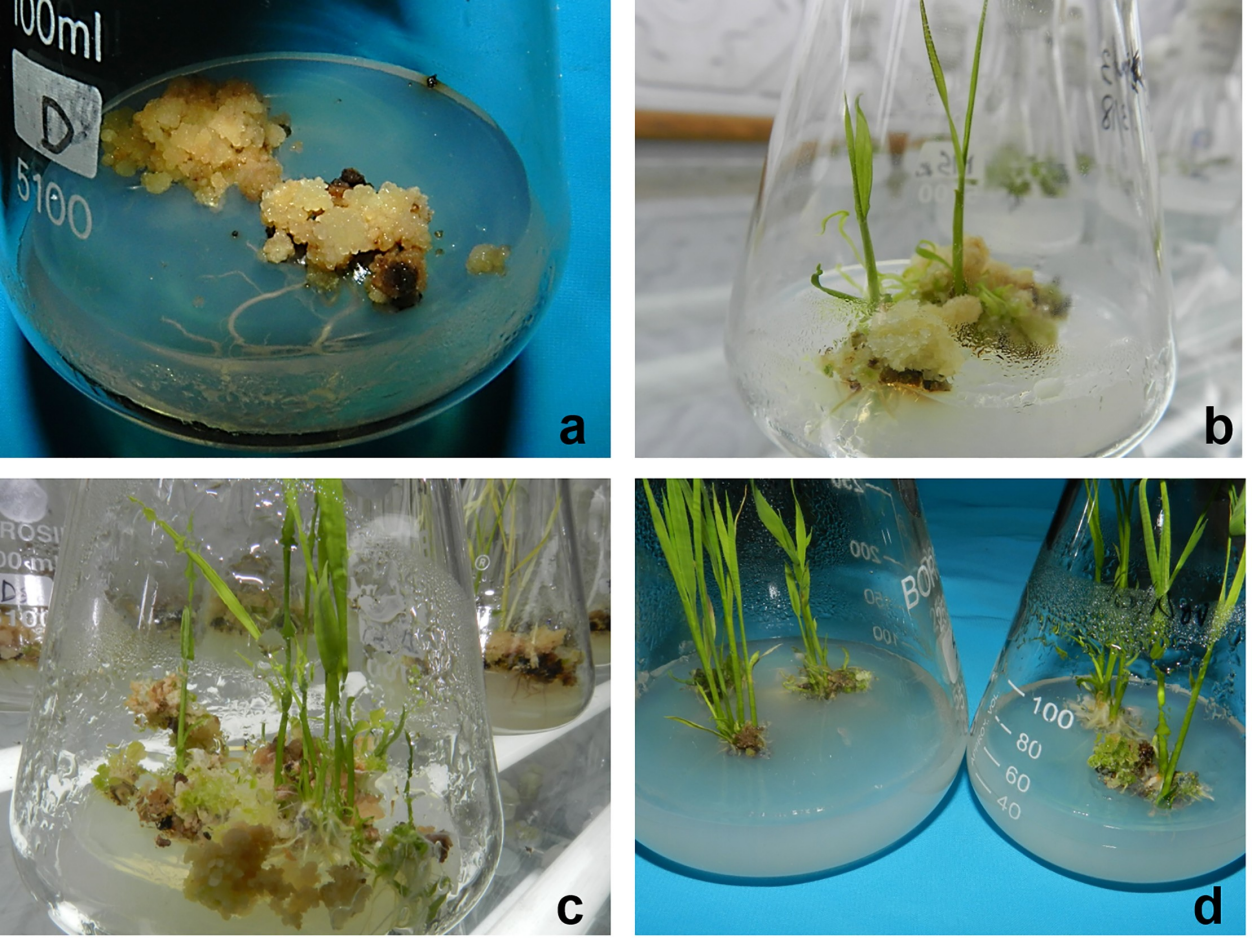
Green plant regeneration from the pollen derived calli on regeneration media. a: Development of roots (combination B), b: Development of shoots (combination C), c: Development of roots and shoots (combination D); d: Sub-culture of individual plantlets.

The green spots started appearing on calli after 10–14 days post inoculation (of calli) and differentiated into green shoots after another 10 days (Fig 3). ANOVA revealed significant mean squares for CP, BES and CP x BES calculated across parents and F_1_. The effect of CP_4_ and CP_6_ both equally enhanced green plant regeneration, while CP_2_ and CP_8_ were least effective and significantly different. Similarly, the main effect of BES was significant at P<0.05 with BES_7-10_ better than BES_11-13_ followed equally by BES_4-6_ and BES_>13_ which showed significant difference among themselves in F_1_. A different trend was observed for main effect of cold in K-332 with maximum shoot regeneration observed in CP_6_ followed by CP_4_ which varied significantly at P<0.05. CP_2_ and CP_8_ though were least effective and showed non-significant difference among themselves. The main effects for BES followed the same trend as in F_1_. In GS-88, CP_4_ and CP_6_ did not vary significantly and showed better regeneration compared to CP_2_ and CP_8_. BES_7-10_ showed better regeneration potential followed by BES_11-13_, BES_4-6_ and BES_>13_. The maximum green plant regeneration was observed for calli originated from the anther that was cold pretreated for 4 days (CP_4_) and collected from the boot with BES of 7–10 inches in all the three genotypes. Green plant regeneration frequency varied from 0% (CP_2_BES_4-6_) to 60.6% (CP_4_BES_7-10_) in F_1_s, 0% (CP_2_BES_7-10_) to 47.7% (CP_2_BES_7-10_) in GS-88 and 0% (CP_2_BES_4-6_) to 47.7% (CP_4_BES_7-10_) in K-332 (Fig 4).

Total regeneration is an inclusive term which refers to regeneration of both green and albino plants. ANOVA revealed that variations due to main effects of CP and BES were significant with no interaction effect across genotypes. CP_6_ and CP_4_ were statistically similar and showed better regeneration than CP_8_ and CP_2_. Likewise, BES_7-10_ and BES_11-13_ were similar and better than BES_>13_ and BES_4-6_ in both genotypes and F_1._ Total regeneration was obtained in a range of 0% (CP_2_BES_4-6_) to 82.7% (CP_6_BES_7-10_) in F_1_, 0% (CP_2_BES_4-6_) to 68.2% (CP_6_BES_7-10_) in K-332 and 0% (CP_2_BES_4-6_) to 54.5% (CP_8_BES_7-10_) in GS-88. CP and BES produced marked effect on number of shoots emerged per green calli.

The main effect of CP and BES (P<0.001) as well as their interaction (P<0.01) were significant for number of shoots/green calli. Maximum number of shoots/green calli was observed in CP_6_BES_7-10_ in both genotypes and F_1._ The main effects of CP_4_ and CP_6_ was statistically different and also better than CP_8_ and CP_2_. Similarly, BES_7-10_ and BES_11-13_ produced more number of shoots per calli than BES_>13_ and BES_4-6_ in both the parents and F_1._ A range of 0% (CP_2_BES_4-6_) to 531.8% (CP_6_BES_7-10_) was observed in F_1,_ 0% (CP_2_BES_4-6_) to 513.6% (CP_6_BES_7-10_) in K-332 and 0% (CP_2_BES_4-6_) to 381.8% (CP_6_BES_7-10_) in GS-88.

Albinism is widely reported phenomenon in anther culture of rice and remains a formidable obstacle in development of doubled haploids. Some of the calli instead of forming green shoots induced white shoot like structure which subsequently developed in to albino plants. Several factors including CP and BES affect the frequency of albinos. ANOVA revealed that variance due to CP (P<0.01) and BES (P<0.001) was significant but the CP × BES interactions were not significant in F_1_ and both parents. It was observed that CP_6_ and CP_4_ were similar and better than CP_8_ and CP_2_. BES_11-13_ and BES_7-10_ showed non-significant difference among themselves and are better than BES_>13_ and BES_4-6_ in both genotypes and F_1._ In variety K-332, CP_6_ was better than other levels of the treatment. In GS-88, however, CP_8_ was found to be the most effective. The highest frequency of albinos was reported in calli induced from anthers incubated at 4°C for 8 days derived from panicles which bear BES of 7–10 inches between flag leaf and penultimate leaf (CP_8_BES_7-10_) in GS-88 and F_1_ that was different what was observed for K-332 where highest albino frequency was observed in CP_6_BES_11-13_. The albino regeneration was 12.1% in CP_4_BES_7-10_ and 35.2% in CP_6_BES_7-10_ for F_1_. In K-332, 7.6% (CP_4_BES_7-10_) and 24.2% (CP_6_BES_7-10_) albinos were recorded compared with 6.8% (CP_4_BES_7-10_) and 6.1% (CP_6_BES_7-10_) in case of the genotype GS-88.

### Analysis of differential genotypic response under anther culture

Since uniform tissue culture conditions may possibly exhibit varied response from genotypes, contrast was sought across K-332, GS-88 and F_1_. Highly significant (P<0.01) mean squares (Group MS) were recorded across genotypes for the parameters, calli induction, green plant regeneration, total regeneration, number of shoots/ plant and albino produced. Within genotype, response across plants was uniform with non-significant differences. Single degree of freedom comparisons were drawn between GS-88 Vs F_1_ and K-332 Vs F_1_ which showed highly significant mean squares at P (< 0.05) for all the traits (Table 4). F_1_ expressed improved androgenesis response over corresponding parents.

**Table 4:** Group balanced ANOVA across the set of genotypes for calli induction and regeneration potential.

Source of variation	d.f	Calli induced	Albino produced	Green plant regeneration	Total regeneration	No. of shoots/plant
**Replication MS**	10	0.24	1.03	1.11	1.86	2.28
**Group MS**	2	1.71 **	0.32 **	0.66 **	1.28 **	0.81 **
**Error (a) MS**	20	0.02	0.06	0.05	0.08	0.10
**F**_**1**_ **MS**	15	1.54 **	2.08 **	2.60 **	4.58 **	5.73 **
**K-332 MS**	15	0.68 **	1.38 **	2.10 **	3.76 **	5.017 **
**GS-88 MS**	15	0.84 **	1.73 **	1.89 **	3.55 **	5.91 **
**F**_**1**_ **Vs K-332**	1	5437.32 **	5102.90 **	4728.79 **	15065.57 **	10189.28 **
**F**_**1**_ **Vs GS-88**	1	6140.82 **	5843.34 **	5124.31 **	16640.52 **	12024.81 **
**Error (b) MS**	448	0.06	0.40	0.32	0.47	0.72

#### Evaluation for agronomic traits

**Fig 5:**
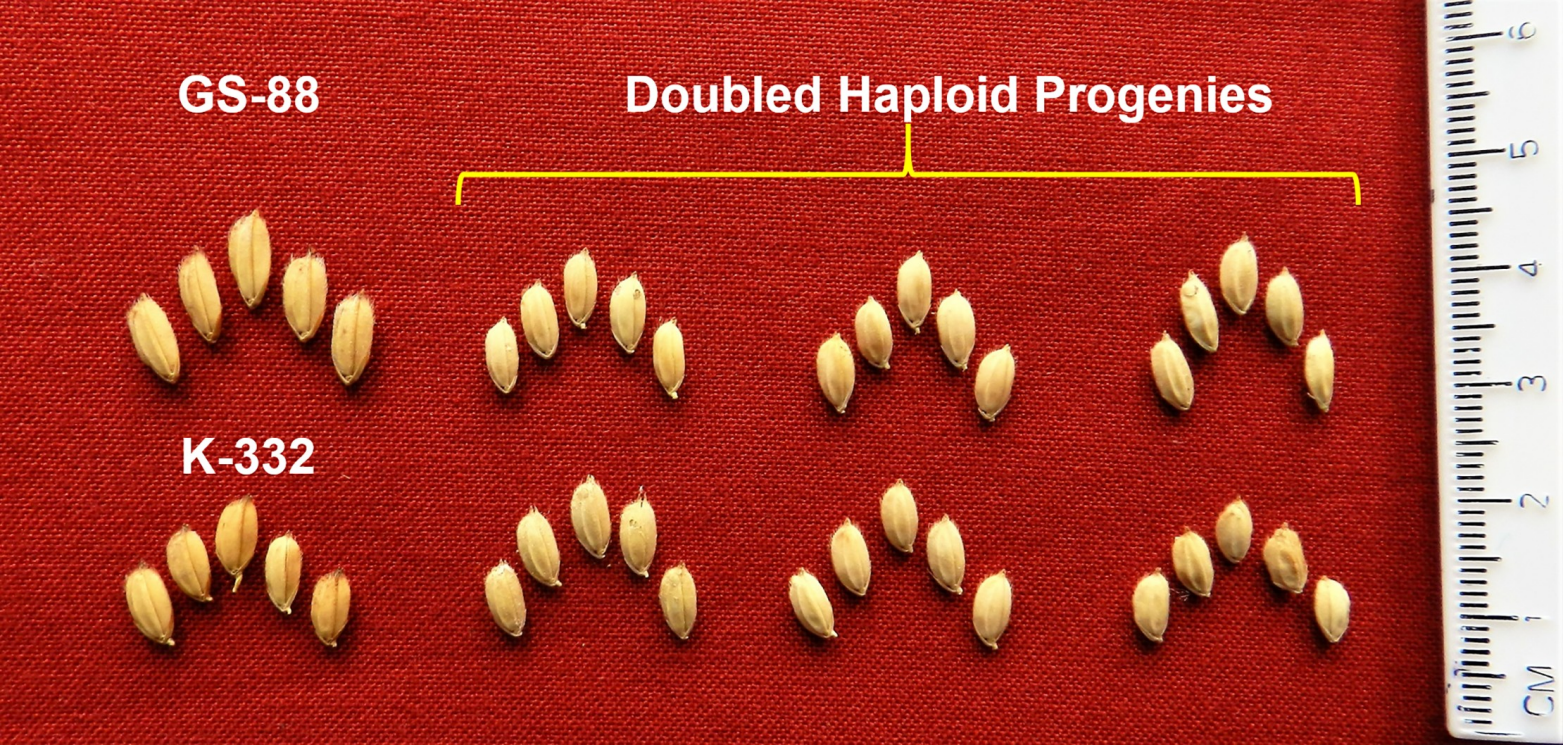
Variation for grain type in doubled haploid progenies derived from K-332 × GS-88.

The DHs were hardened and grown up to maturity in the green house (S1 Table; S4 and S5 Figs). The plants recorded the number of spikelets per panicle and spikelet fertility values within the range of 70–139 and 60.0–85.8%, respectively. The grain length across individuals ranged from 4.5–8.9 mm (S1 Table, Fig 5). The progenies derived from first generation pollen derived plants were raised and recorded plant height in the range of 74.1 to 115.8 inches. The effective tillers per plant ranged from 10.1 to 26.0. Panicle length and spikelets per panicle ranged from 25.0 to 116.0 and 2.0 to 52.6, respectively. The grain yield per plant was recorded within the range of 13.3 to 69.0 g per plant (S2 Table).

## Discussion

The number of exogenous and endogenous factors and their interaction can influence the rates of *in vitro* calli induction and regeneration of which genotype and composition of the culture medium are two important factors. N6 media supplemented with tryptophan and cysteine was initially used as it has been reported to improve calli induction and regeneration rates in recalcitrant basmati rice cultivars [15]. The maltose has been used as a preferred carbon source in tissue culture media and also has previously been found suitable in rice anther culture [16–18]. Maltose is degraded slowly in the culture media and yields only glucose upon hydrolysis. Contrarily, sucrose is metabolized rapidly into glucose and fructose and the latter is known to have a detrimental effect on calli induction in wheat [19, 20]. The dusted anthers turned light brown in color and then swelled in size with absence of calli induction for all the six genotypes studied even after three months of anther inoculation on N6 media. The androgenic response is a genetically determined trait that displays quantitative inheritance. The variation in androgenic response among cultured anthers of different genotypes has been attributed to genetic sources such as additive, non-additive, dominant effects and cytoplasmic effects [7]. N6 media contain inorganic nitrogen in the form of NO^3-^ (3535 mg/l) and NH_4_^+^ (463 mg/l) ions which is generally preferred by japonica rice cultivars. Even though the recalcitrance can be activated by manipulation of non-genetic factors such as media, significant improvement in the calli induction may not be achieved in recalcitrant genotypes since calli induction is largely under genetic control [7]. Our results suggest that N6 media which is used for rice anther in general, may not be specific and suitable for the genotypes in our case to effect callus initiation. Reiffers and Freire [21] observed less than 1% calli induction (0.2–0.7) in two *japonica × japonica* and *indica× japonica* hybrids respectively when inoculated on N6 media. Similarly no calli induction was observed in CH2 Double dwarf variety, when 10,500 anthers were plated on N6 media [22]. Khatun et al., [23] tested 20 genotypes for assessment of their androgenic response, out of which only 5 responded well. The genotypes reported better androgenic response on SK-3 media than N6 media. These results support the hypothesis that while N6 media has been established as a media of choice for developing DHs in both japonica (high efficiency) and indica (low efficiency) rice varieties, the reason behind non-effective role of N6 media in inducing calli in the genotypes under study may be due to genotype specific culture media requirements in rice anther culture. Genotype specific culture media requirements have been reported in rice anther culture system underscoring an inescapable reality that though media components bring an appreciable improvements in anther culture, culture media requirements and optimization depend upon individual genotype [7, 24]. Therefore, a new media with different combinations of macro and micro nutrients which can effectively activate recalcitrant genes and may be useful as an alternative or adjuvant to N6 media. Media components have a visible effect on the induction of callusing in anther culture as indicated by a number of experiments [22, 25]. We report an alternate media namely ‘N6_M_ media’ that was formulated by overall change in the concentration of various macro and micro nutrients_._ The concentration of NO_3_ (3250 mg/l) and NH_4_ (300 mg/l) ions was reduced and concentration of various micro nutrients was increased in comparison N6 media. The lower concentration of ammonia has been found beneficial for anther culture of rice and other cereals like wheat and barley [26, 27]. However, the overall ratio of NO_3_: NH_4_ was increased in this case which also has been reported to positively influence anther culture response in rice [25]. Microelements play an important and sometimes decisive role in the anther culture [5]. Anthers from F_1_ (K-332 × GS-88) were implanted on N6_M_ media after proper pretreatment. After 15 days of incubation at 25±2°C in total darkness, anthers first turned light brown and then swelled up followed by bursting out of calluses from the middle of anthers. The callusing frequency was 2.2% and the calli obtained were compact and light yellow in color. The culturing of pollens along with anthers improves the callusing rates. The release of certain chemicals during anther senescence helps in the pollen division and embryo development. Moreover, the anther walls senescence and turn brown, thus minimizing the chance of callus induction from cells of anther wall [28]. Raina and Zapata [25] observed higher calli induction rates for indica varieties on SK-I media which uses low concentration of ammonium ions. Though, better calli induction rates were observed for indica variety *Kurulu Thuda* on N6 media as compared to SK-I media [7]. The results from this study emphasize on the possibility of enhancement of calli induction rates by manipulation of media composition.

We observed that only 2–3 mm F_1_ androgenic calli showed better regeneration potential compared with much bigger calli. That may be explained as prolonged exposure of pollen derived calli to high concentration of 2,4-D have been reported to affect the frequency of regenerated plants in cereal crops [29, 30]. Since, 2,4-D and NAA, both are synthetic auxins, therefore, NAA formed the vital ingredient of regeneration media. Plant growth regulators play a vital role in determining the success of plant regeneration in anther culture. The combinations of hormone type and hormonal concentration and their interactions are known to influence *invitro* regeneration of plants [18]. NAA as auxin source and two cytokinins (BAP and Kinetin) were studied for their effect on the haploid regeneration rates from pollen derived calli. These three hormones were used to formulate four different combinations, A, B, C and D based on their relative concentrations. Of these, combination B promoted root development in calli while combination C initiated the proliferation of shoots. The combination D which comprised of BAP (2 mg/l), kinetin (1 mg/l) and NAA (1 mg/l) was effective in regeneration of both roots and shoots and therefore, was chosen for further investigation.

Cold shock (CP) is the commonly administered stress treatment that promotes androgenesis in rice and in other cereals. The duration and time of application of cold shock plays an important role in enhancing the calli induction rates. Cold pre-treatment is reported to arrest the gametophytic development of microspores and guide the microspores towards sporophytic developmental pathway [31]. The mechanism which guides this transition of microspores from gametophytic to sporophytic development remains unexplained. However, cold stress is reported to slow down the degradation process of anther walls, starvation of microspores disconnected from tapetum, promote symmetric division of pollen grains and total content of free amino acids and small heat shock protein (HSP) [7, 32]. The release of free amino acids might be conducive to adaptation of microspores to metabolic changes of androgenesis while as HSPs might protect microspores against freezing stress. Secondly, the stage of panicle emergence (BES) is an important factor in androgenic response as culturing of advance boot stages may be difficult due to their commitment to differentiation into a male gamete. The two pre-treatments of harvested anthers are related to each other as microspores are responsive to cold stress at a particular stage of their development which may recourse their developmental pathway from gametophyte to sporophyte [33]. Anthers containing pollens at mid to late uninucleate stage are the most responsive and suitable for anther culture [6, 7]. BES measured as the length between the base of the flag and penultimate leaf of the productive tiller has reportedly shown close association with pollen development stage [34] and has been suggested to be a morphological indicator for determining the time of boot collection [35, 36]. The callus induction rate varied in response to CP and BES treatments. Out of the 16 factorial combinations, CP_6_ and BES_7-10_ was found to be uniformly efficient across parents (9.5% in GS-88 and 6.7% in K-332) and F_1_ (27.6%). Main effect of BES was significant with BES_7-10_ better than BES_11-13_ which was in agreement with previous studies [37]. Khatun et al., [23] reported highest embryo production from anthers cold pretreated at 4°C for 5 day induction period. The extended periods of cold pre-treatment were found to negatively affect calli induction rate which has been supported by Lenka and Reddy and Cristoffanini et al., [38, 24] Similarly, Trejo-Tapia et al., [39] also reported cold pretreatment at 4°C for extended periods (14 and 21 day period) inhibitory for androgenesis. In addition, F_1_ hybrid showed higher response for calli induction than parents. The higher response of F_1_ towards calli induction may be due to additive effect of anther culture responsive genes from two parents. Similar response was obtained for F_1_ hybrids in study conducted by Bishnoi et al., [22]. Under these treatments, the calli obtained were compact, creamy white and nodular in appearance and resulted in better green plant regeneration.

### Shoot regeneration

A high green plant regeneration of 60.6% in F_1_, 47.7% in both GS-88 and K-332 was recorded. Most of the calli derived from anthers were compact in texture and creamy white in colour which subsequently developed into green plants and a few regenerated into albino plants. However, none of the friable calli developed into green shoots and had poor regeneration ability. Maximum shooting percentage of 531.8 (F_1),_ 513.6 (K-332), 381.8% (GS-88) was observed for CP_6_BES_7-10_ treatment. Besides due to appropriate stage of collection and incubation period, the overall high rates of green plant regeneration in our study might have been because the materials belonged to japonica which are reported to show high regeneration rates. The green plantlets derived from the embryogenic callus of indica rice (Suphanburi, Binnatoe, and BR-7) were reported to be low (0.1–10%), while those derived from japonica rice (Notohikari and Taipei-309) were higher at 8.9–62% [16, 40]. Another reason for high green plant regeneration can be attributed to the use of low 2,4-D concentration (0.5 mg/l) in the calli-induction medium. Significant genotypic differences for green plant regeneration was observed among the genotypes and resulting F_1_s_._ In this study, F_1_ showed better green plant regeneration compared to both parents.

Albinism has also been reported in rice and remains a formidable obstacle in development of doubled haploids via anther culture [41, 42]. The highest frequency of albinos was reported in calli induced from anthers incubated for eight days and low albino regeneration has been observed for calli cold incubated for shorter durations of time (CP_4_BES_7-10)._ This implies that cold pre-treatment for longer durations can have negative impact on pollen and can lead to loss of chlorophyll which ultimately results in albino generation. Torp and Anderson [43] suggested that cold stress for longer duration make the plants fight their own plastids with antibiotic-like compounds. Large-scale deletions in some plastid genomes of the albino plants derived from the anther culture of japonica & indica hybrids and absence of such deletions in green regenerates [44] validate the statement.

The wide range of values for agronomic traits like plant height, panicle length, effective tillers per plant, spikelets per panicle and grain yield indicates the high variability within DH population. The trait means at population level centered within the parental range and therefore, indicate normal diploidy of plants which may be validated through karyotyping for selected number of plants in future studies.

## Conclusion

In conclusion, a modified N6_M_ media was developed to reverse the recalcitrance of the genotypes under study for androgenesis. We also report the optimization of the duration of cold pre-treatment (CP: 4–6 days) for efficient callus initiation and regeneration. We further report the optimal stage of panicle development (BES: 7 inches) for boot harvesting. The improved media conditions and optimal strategy for anther harvesting and pre-treatment process before tissue culture can improve the androgenisis potential in rice as well as other cereals. In this study, a total of 207 DH lines were generated which can serve as a valuable resource for genomics and mapping studies.

## Supporting information

S1 Fig Callus culture on regeneration media. (PDF)

S2 Fig Boot Emergence Stage (BES) for collection of anthers. BES_4-6_: 4–6, BES_7-10_: 7–10, BES_11-13_: 11–13 and BES_>13_: >13 inches.
(PDF)

S3 Fig Polymorphism survey between GS-88 and K-332 using SSR markers distributed on different chromosomes. M: 100 bp DNA ladder (Thermo Fisher Scientific, New Delhi, India); Left well: GS-88; Right well: K-332.
(PDF)

S4 Fig The process of hardening of anther culture derived plantlets. (PDF)

S5 Fig Panel of doubled haploid progenies developed through anther culture. (PDF)

S1 Table Grain attributes of doubled haploids produced from K-332 x GS-88. (PDF)

S2 Table Evaluation of DH progenies for agronomic performance under field conditions. (PDF)

S1 File Main and interaction effects pronounced by Cold Pre-treatment (CP) and Boot Emergence Stage (BES) on plant regeneration. (XLSX)

S1 Raw images (PDF)
