# Supplementary material for: Improved protocol for efficacious in vitro androgenesis and development of doubled haploids in temperate japonica rice
Source: PLoS One. 2020 Nov 2;15(11):e0241292. doi: 10.1371/journal.pone.0241292 (PMC7605686; doi:10.1371/journal.pone.0241292)
Supplement: S1 Fig — (PDF) [file pone.0241292.s001.pdf]

S1 Fig. Callus culture on regeneration media

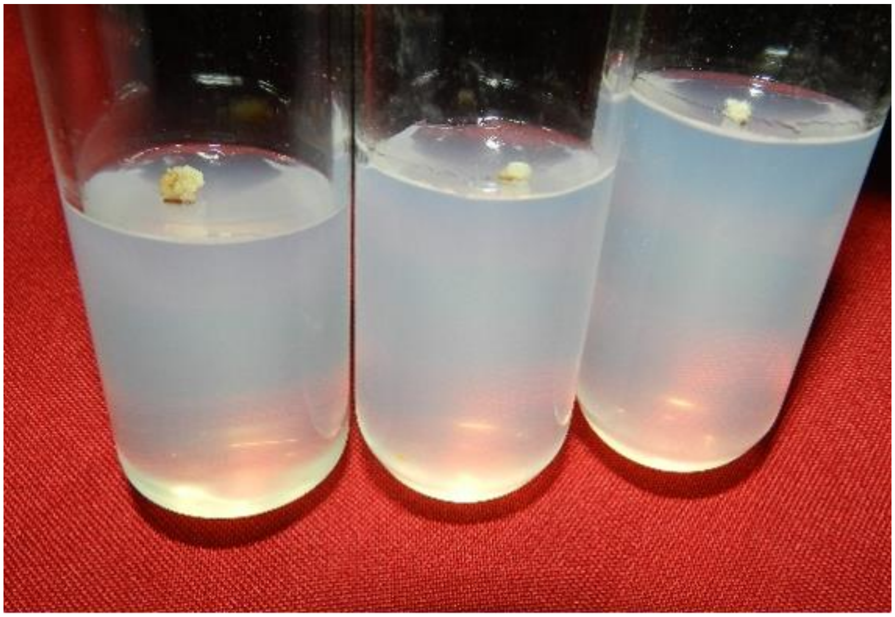

Primary culture of 2-3mm calli for regeneration

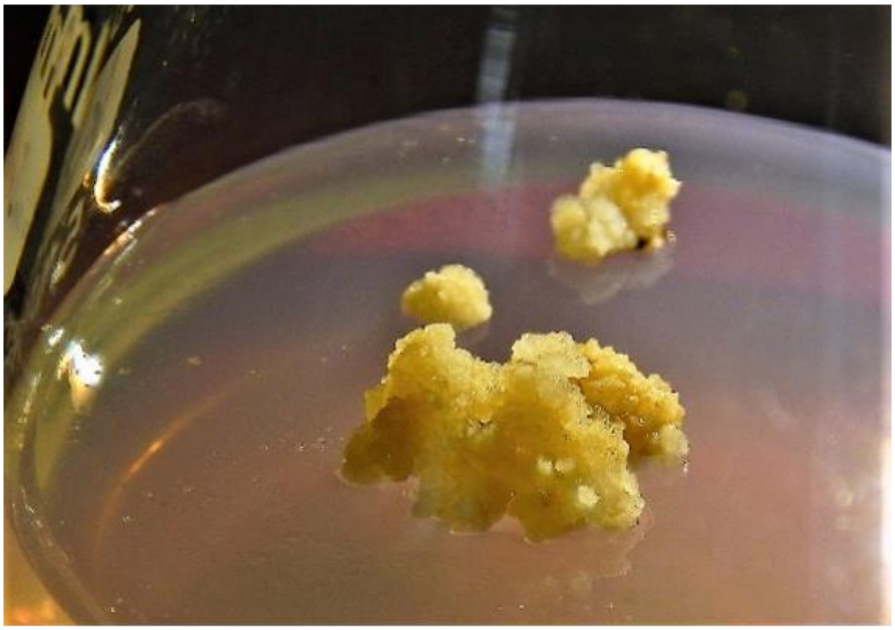

Sub-culture
