# Supplementary figures and images for: Improved protocol for efficacious in vitro androgenesis and development of doubled haploids in temperate japonica rice
Source: PLoS One. 2020 Nov 2;15(11):e0241292. doi: 10.1371/journal.pone.0241292 (PMC7605686; doi:10.1371/journal.pone.0241292)

**S2 Fig. Boot emergence stage (BES) for collection of anthers**

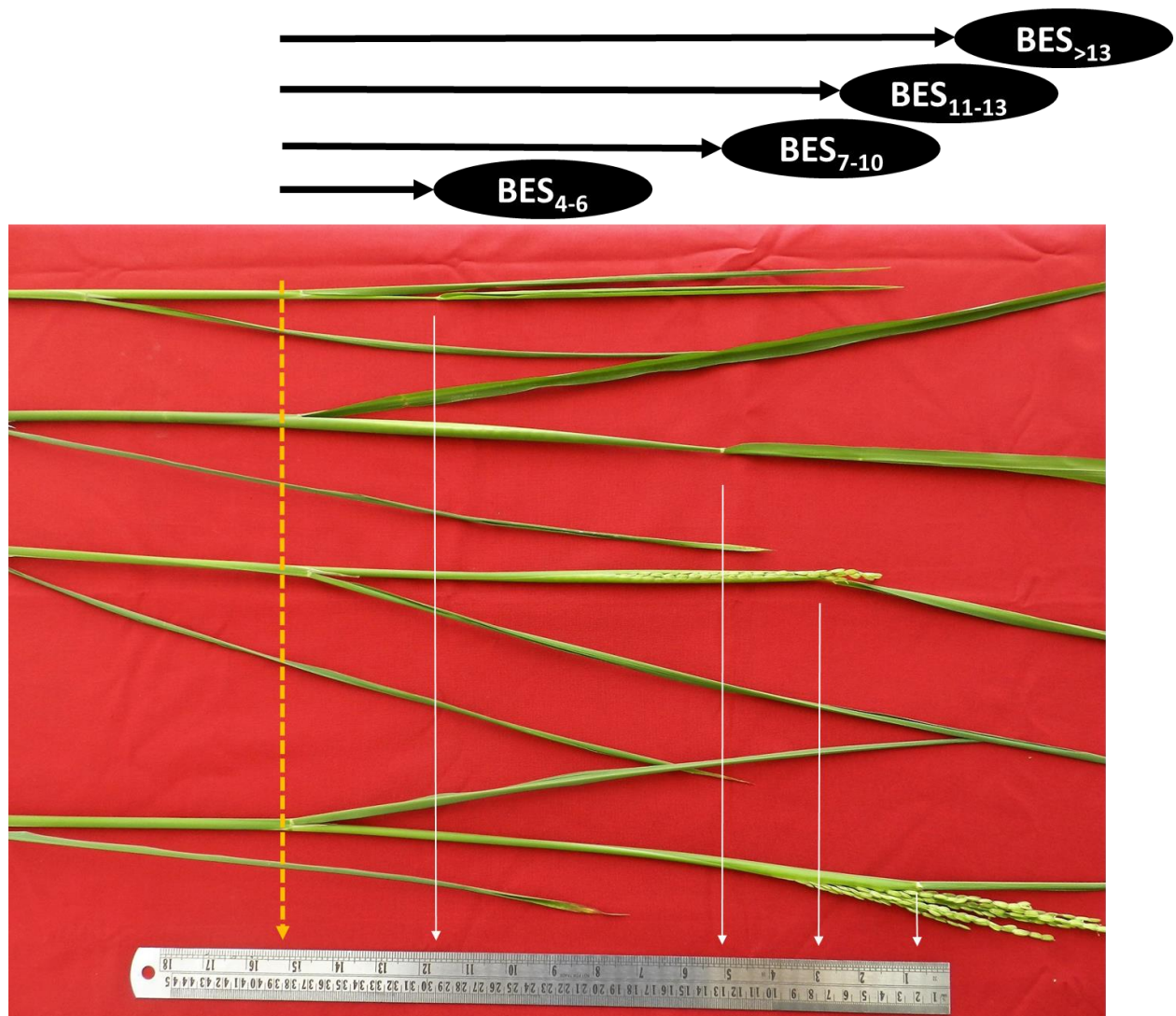

$BES_{4-6}$ : 4-6,  $BES_{7-10}$ : 7-10,  $BES_{11-13}$ : 11-13 and  $BES_{>13}$ : >13 inches

Supplement: S2 Fig — BES4-6: 4–6, BES7-10: 7–10, BES11-13: 11–13 and BES>13: >13 inches. (PDF) [file pone.0241292.s002.pdf]

**S4 Fig. The process of hardening of anther culture derived plantlets**

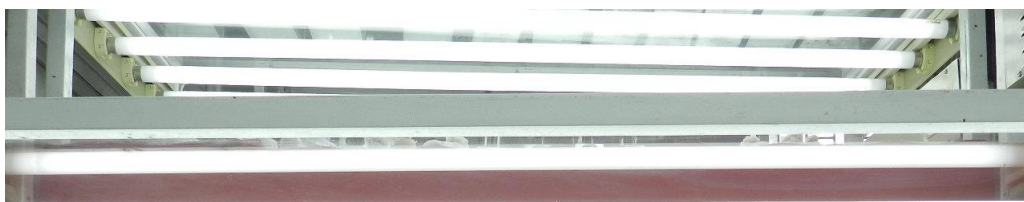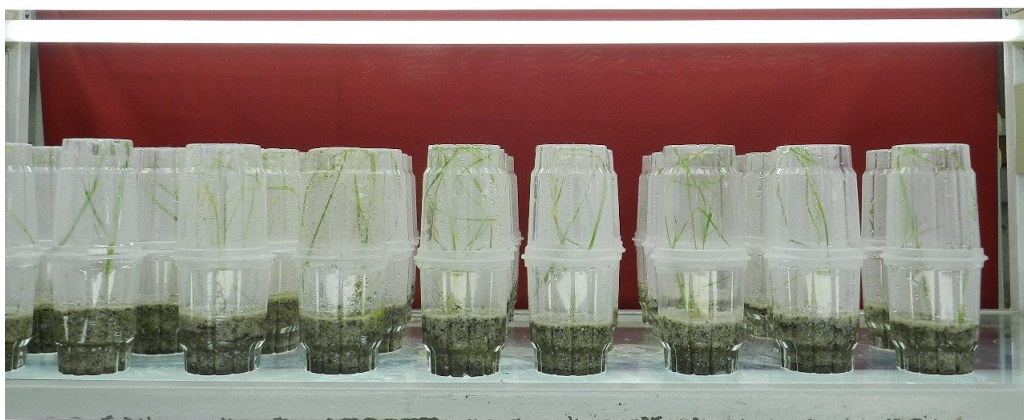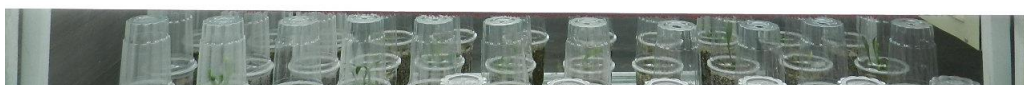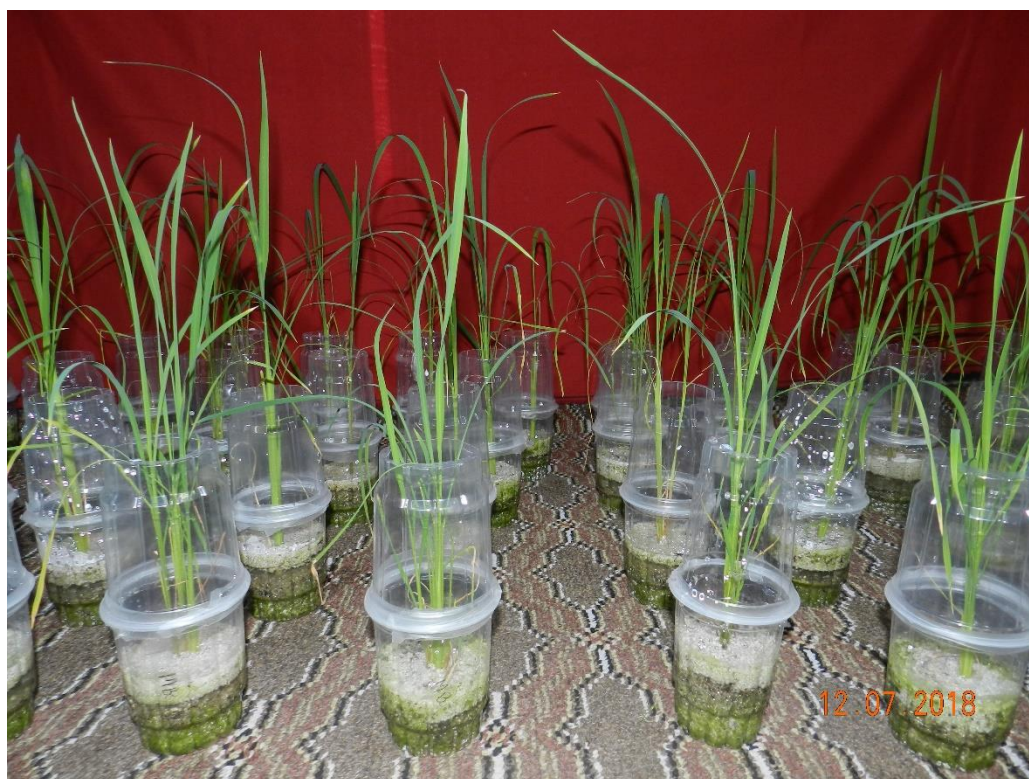

Supplement: S4 Fig — (PDF) [file pone.0241292.s004.pdf]

**S5 Fig. Panel of doubled haploid progenies developed through anther culture**

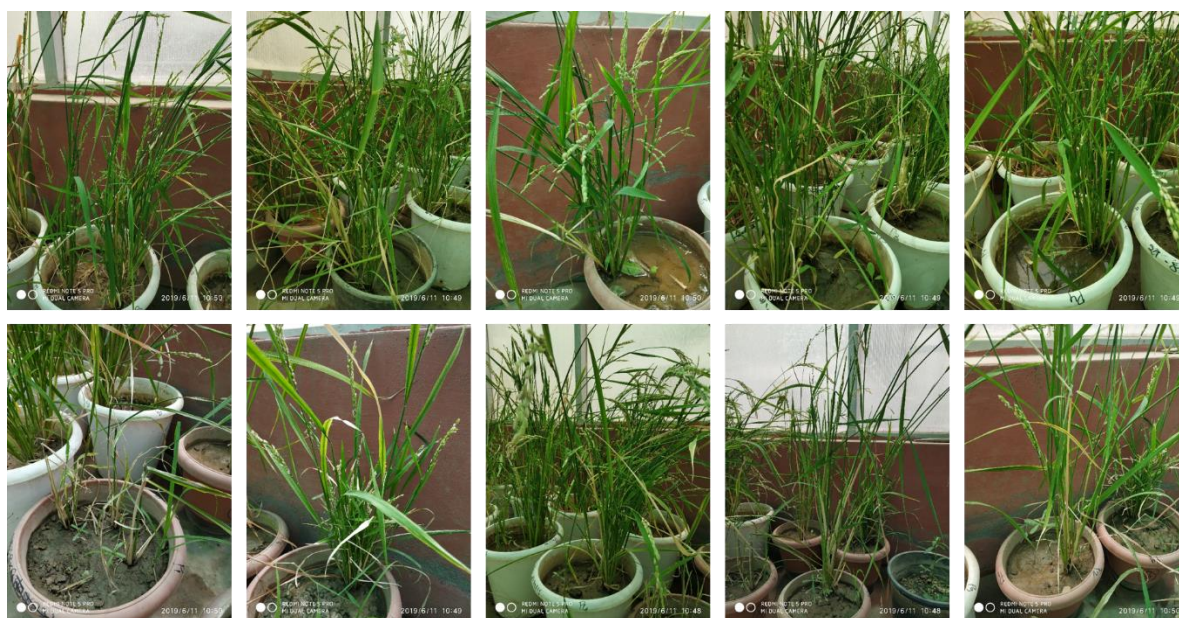

Supplement: S5 Fig — (PDF) [file pone.0241292.s005.pdf]
