# Supplementary material for: Improved protocol for efficacious in vitro androgenesis and development of doubled haploids in temperate japonica rice
Source: PLoS One. 2020 Nov 2;15(11):e0241292. doi: 10.1371/journal.pone.0241292 (PMC7605686; doi:10.1371/journal.pone.0241292)
Supplement: S3 Fig — M: 100 bp DNA ladder (Thermo Fisher Scientific, New Delhi, India); Left well: GS-88; Right well: K-332. (PDF) [file pone.0241292.s003.pdf]

S3 Fig. Polymorphism survey between GS-88 and K-332 using SSR markers distributed on different chromosomes

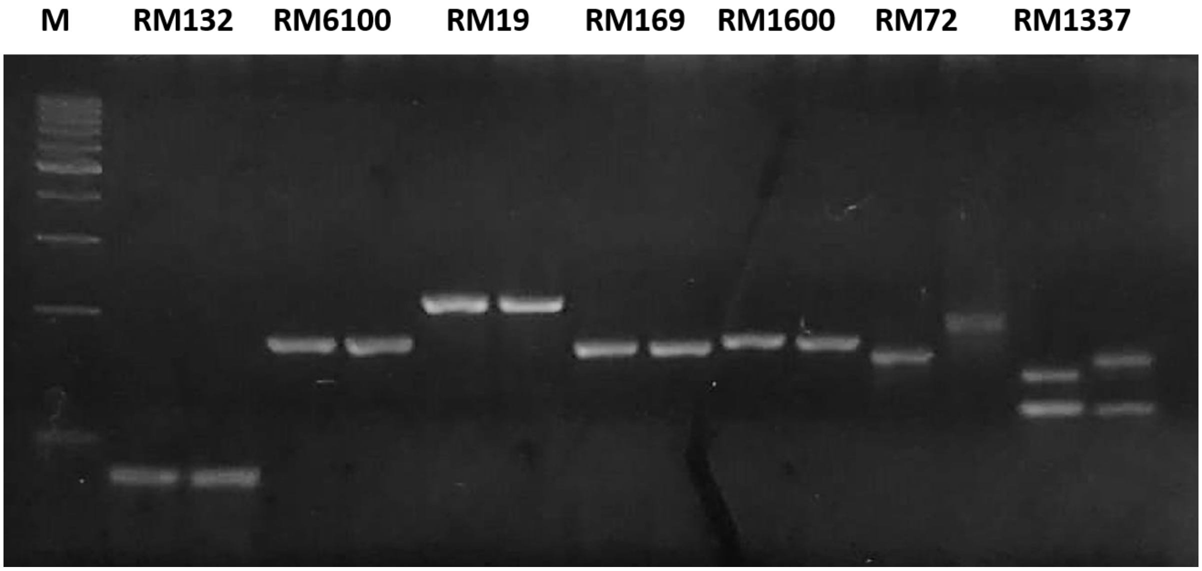

M: 100 bp DNA Ladder (Thermo Fisher Scientific, New Delhi, India); Left well: GS-88; Right well: K-332
