# Supplementary material for: Improved protocol for efficacious in vitro androgenesis and development of doubled haploids in temperate japonica rice
Source: PLoS One. 2020 Nov 2;15(11):e0241292. doi: 10.1371/journal.pone.0241292 (PMC7605686; doi:10.1371/journal.pone.0241292)
Supplement: S1 Table — (PDF) [file pone.0241292.s006.pdf]

S1 Table. Grain attributes of Doubled haploids produced from K-332 x GS-88

Plant#ID	Grain length (mm)	Number of spikelets/ panicle	Spikelet fertility (%)
SKUA-DH-1	7.1	117	78.4
SKUA-DH-2	6.9	112	71.4
SKUA-DH-3	8.6	94	67.0
SKUA-DH-4	6.3	90	68.4
SKUA-DH-5	5.4	115	64.2
SKUA-DH-6	8.9	139	63.5
SKUA-DH-7	4.7	106	63.2
SKUA-DH-8	4.9	73	65.0
SKUA-DH-9	8.8	72	70.4
SKUA-DH-10	6.7	110	65.3
SKUA-DH-11	8.7	107	64.5
SKUA-DH-12	8.9	98	84.1
SKUA-DH-13	7.9	109	80.4
SKUA-DH-14	8.6	119	81.0
SKUA-DH-15	6.8	84	72.1
SKUA-DH-16	8.3	97	68.9
SKUA-DH-17	8.5	109	74.9
SKUA-DH-18	8.9	74	61.5
SKUA-DH-19	5.5	107	71.4
SKUA-DH-20	6.8	85	72.6
SKUA-DH-21	5.2	97	63.7
SKUA-DH-22	6.5	93	66.8
SKUA-DH-23	8.0	138	76.5
SKUA-DH-24	7.6	97	80.2
SKUA-DH-25	4.7	85	71.1
SKUA-DH-26	5.0	75	81.1
SKUA-DH-27	5.8	78	69.0
SKUA-DH-28	6.8	114	75.6
SKUA-DH-29	7.0	109	82.6
SKUA-DH-30	8.1	96	67.6
SKUA-DH-31	6.6	138	76.2
SKUA-DH-32	5.6	104	64.1
SKUA-DH-33	6.1	97	69.7
SKUA-DH-34	7.1	75	72.4
SKUA-DH-35	6.4	93	61.1
SKUA-DH-36	7.0	77	81.8
SKUA-DH-37	7.8	80	64.2
SKUA-DH-38	6.2	111	80.1
SKUA-DH-39	8.9	122	82.0

SKUA-DH-40	8.2	81	66.3
SKUA-DH-41	5.4	124	77.3
SKUA-DH-42	4.6	128	85.2
SKUA-DH-43	8.7	92	60.0
SKUA-DH-44	8.9	125	75.4
SKUA-DH-45	7.3	73	74.2
SKUA-DH-46	7.4	138	63.4
SKUA-DH-47	8.2	134	81.6
SKUA-DH-48	8.1	117	64.1
SKUA-DH-49	6.1	110	77.0
SKUA-DH-50	8.0	84	84.5
SKUA-DH-51	5.3	108	71.5
SKUA-DH-52	5.3	106	81.2
SKUA-DH-53	5.3	88	83.4
SKUA-DH-54	8.2	78	78.6
SKUA-DH-55	5.6	106	77.9
SKUA-DH-56	8.7	132	68.5
SKUA-DH-57	6.3	93	60.4
SKUA-DH-58	5.9	85	62.5
SKUA-DH-59	8.5	77	68.5
SKUA-DH-60	8.1	135	73.9
SKUA-DH-61	8.2	111	82.7
SKUA-DH-62	6.4	121	66.6
SKUA-DH-63	8.4	137	77.9
SKUA-DH-64	5.9	80	63.5
SKUA-DH-65	7.9	87	71.8
SKUA-DH-66	5.3	93	73.6
SKUA-DH-67	7.7	126	84.6
SKUA-DH-68	6.9	115	60.9
SKUA-DH-69	4.9	108	76.0
SKUA-DH-70	7.2	84	73.5
SKUA-DH-71	5.3	108	61.1
SKUA-DH-72	4.8	130	74.4
SKUA-DH-73	7.9	81	67.7
SKUA-DH-74	5.4	120	61.9
SKUA-DH-75	5.1	116	81.5
SKUA-DH-76	5.1	74	80.4
SKUA-DH-77	7.6	125	65.8
SKUA-DH-78	7.9	84	74.6
SKUA-DH-79	5.6	131	62.4
SKUA-DH-80	5.4	120	78.4

SKUA-DH-81	7.3	94	71.4
SKUA-DH-82	8.4	78	82.1
SKUA-DH-83	5.9	70	72.9
SKUA-DH-84	8.2	97	83.6
SKUA-DH-85	8.8	116	63.1
SKUA-DH-86	6.3	108	76.4
SKUA-DH-87	7.8	84	60.9
SKUA-DH-88	5.8	86	84.5
SKUA-DH-89	7.2	71	68.4
SKUA-DH-90	7.4	117	74.8
SKUA-DH-91	6.3	100	69.8
SKUA-DH-92	8.5	127	85.4
SKUA-DH-93	5.5	101	67.0
SKUA-DH-94	7.2	102	60.6
SKUA-DH-95	6.8	79	78.0
SKUA-DH-96	6.5	127	73.3
SKUA-DH-97	6.6	76	70.0
SKUA-DH-98	6.7	132	74.0
SKUA-DH-99	6.0	105	63.8
SKUA-DH-100	6.4	107	62.4
SKUA-DH-101	6.6	131	76.3
SKUA-DH-102	5.4	84	85.0
SKUA-DH-103	5.3	78	63.4
SKUA-DH-104	8.9	115	60.8
SKUA-DH-105	4.7	134	73.3
SKUA-DH-106	8.7	119	84.8
SKUA-DH-107	8.4	131	61.9
SKUA-DH-108	6.6	122	76.0
SKUA-DH-109	8.5	115	62.9
SKUA-DH-110	8.3	87	62.2
SKUA-DH-111	6.3	87	83.6
SKUA-DH-112	4.7	133	75.2
SKUA-DH-113	7.1	135	77.7
SKUA-DH-114	8.8	132	67.3
SKUA-DH-115	8.7	80	82.4
SKUA-DH-116	6.7	136	83.4
SKUA-DH-117	5.8	79	83.1
SKUA-DH-118	7.4	110	85.8
SKUA-DH-119	8.8	112	79.8
SKUA-DH-120	5.1	99	83.7
SKUA-DH-121	8.8	102	63.6

SKUA-DH-122	8.6	120	79.7
SKUA-DH-123	5.1	81	77.7
SKUA-DH-124	4.5	111	61.3
SKUA-DH-125	5.5	86	80.0
SKUA-DH-126	5.8	73	80.0
SKUA-DH-127	8.5	86	69.9
SKUA-DH-128	7.2	93	77.9
SKUA-DH-129	4.7	92	78.1
SKUA-DH-130	7.5	108	65.6
SKUA-DH-131	7.1	96	85.6
SKUA-DH-132	6.7	128	68.5
SKUA-DH-133	8.5	93	65.7
SKUA-DH-134	5.3	105	62.5
SKUA-DH-135	4.9	109	64.3
SKUA-DH-136	5.1	107	60.8
SKUA-DH-137	6.1	86	84.6
SKUA-DH-138	5.8	90	81.8
SKUA-DH-139	6.8	75	72.8
SKUA-DH-140	5.7	102	60.7
SKUA-DH-141	7.7	137	72.1
SKUA-DH-142	8.1	119	72.9
SKUA-DH-143	6.8	135	84.6
SKUA-DH-144	4.7	137	80.6
SKUA-DH-145	6.1	95	75.2
SKUA-DH-146	8.9	134	70.0
SKUA-DH-147	8.1	116	67.3
SKUA-DH-148	7.4	101	85.5
SKUA-DH-149	6.2	85	68.7
SKUA-DH-150	8.2	76	63.2
SKUA-DH-151	6.4	85	81.4
SKUA-DH-152	7.2	102	68.5
SKUA-DH-153	5.3	137	72.6
SKUA-DH-154	4.8	130	61.8
SKUA-DH-155	6.2	72	70.2
SKUA-DH-156	6.0	92	64.0
SKUA-DH-157	6.2	88	77.1
SKUA-DH-158	7.4	132	84.3
SKUA-DH-159	8.2	114	83.6
SKUA-DH-160	5.6	103	82.2
SKUA-DH-161	8.0	74	68.1
SKUA-DH-162	7.3	136	71.7

SKUA-DH-163	8.6	136	77.5
SKUA-DH-164	5.7	111	61.3
SKUA-DH-165	8.8	94	76.5
SKUA-DH-166	8.7	84	82.0
SKUA-DH-167	6.6	105	83.8
SKUA-DH-168	6.0	82	75.4
SKUA-DH-169	7.5	135	62.0
SKUA-DH-170	6.6	80	65.9
SKUA-DH-171	4.6	102	67.8
SKUA-DH-172	7.7	87	66.9
SKUA-DH-173	8.6	97	73.2
SKUA-DH-174	6.1	97	80.2
SKUA-DH-175	8.6	138	78.2
SKUA-DH-176	5.7	71	75.1
SKUA-DH-177	5.5	127	80.1
SKUA-DH-178	8.7	121	84.9
SKUA-DH-179	8.1	77	62.6
SKUA-DH-180	4.9	105	66.7
SKUA-DH-181	4.7	97	76.6
SKUA-DH-182	4.5	106	62.6
SKUA-DH-183	8.9	116	78.4
SKUA-DH-184	8.6	79	63.0
SKUA-DH-185	5.6	128	66.0
SKUA-DH-186	5.7	137	85.5
SKUA-DH-187	5.3	103	72.5
SKUA-DH-188	5.2	72	65.6
SKUA-DH-189	7.1	76	70.0
SKUA-DH-190	8.6	111	62.0
SKUA-DH-191	8.3	82	84.8
SKUA-DH-192	7.2	107	64.5
SKUA-DH-193	5.8	113	65.7
SKUA-DH-194	6.5	92	60.1
SKUA-DH-195	7.7	112	79.9
SKUA-DH-196	5.6	88	85.5
SKUA-DH-197	5.7	85	60.4
SKUA-DH-198	5.6	108	76.1
SKUA-DH-199	5.4	133	80.9
SKUA-DH-200	5.3	131	81.9
SKUA-DH-201	8.1	101	85.5
SKUA-DH-202	4.8	89	68.9
SKUA-DH-203	7.6	120	67.3

SKUA-DH-204	6.3	117	68.9
SKUA-DH-205	6.4	86	69.8
SKUA-DH-206	7.7	81	80.8
SKUA-DH-207	5.9	75	66.1
**Mean**	**6.8**	**103.4**	**72.7**
**Range**	**4.5 - 8.9**	**70.0 – 139.0**	**60.0 - 85.8**
**K-332**	**6.3**	**134.5**	**90.5**
**GS-88**	**8.6**	**112.7**	**83.6**
