# Supplementary material for: Improved protocol for efficacious in vitro androgenesis and development of doubled haploids in temperate japonica rice
Source: PLoS One. 2020 Nov 2;15(11):e0241292. doi: 10.1371/journal.pone.0241292 (PMC7605686; doi:10.1371/journal.pone.0241292)
Supplement: S2 Table — (PDF) [file pone.0241292.s007.pdf]

S2_Table. Evaluation of DH progenies for agronomic performance under field conditions

Plant#ID	Plant height (cm)			Number of Tillers per plant			Panicle length (cm)			Number of spikelets/ panicle			Grain yield per plant (g)		
SKUA-DH-1	104.6	±	2.1	15.7	±	0.2	20.5	±	0.5	102.8	±	2.5	38.3	±	2.2
SKUA-DH-2	99.2	±	4.9	23.1	±	0.3	17.9	±	0.5	91.1	±	4.1	52.3	±	1.8
SKUA-DH-3	93.2	±	3.3	10.3	±	0.9	22.5	±	0.4	111.9	±	4.1	27.8	±	2.7
SKUA-DH-4	85.2	±	4.7	10.3	±	0.6	18.9	±	0.3	53.8	±	4.2	13.3	±	2.4
SKUA-DH-5	103.2	±	4.4	25.9	±	0.2	13.5	±	0.0	110.0	±	4.7	68.1	±	1.5
SKUA-DH-6	91.9	±	2.8	22.7	±	0.7	19.3	±	0.1	95.8	±	4.1	52.3	±	3.3
SKUA-DH-7	106.5	±	2.9	25.7	±	0.3	22.7	±	0.0	99.7	±	2.2	61.9	±	1.0
SKUA-DH-8	96.2	±	2.3	20.3	±	0.2	14.8	±	1.0	80.1	±	3.2	40.0	±	2.3
SKUA-DH-9	99.6	±	4.7	18.5	±	0.7	17.9	±	0.3	82.4	±	4.1	36.9	±	2.6
SKUA-DH-10	100.9	±	4.1	18.7	±	0.6	17.9	±	0.1	105.7	±	2.6	47.3	±	3.4
SKUA-DH-11	109.9	±	3.8	11.0	±	0.3	23.2	±	0.5	70.3	±	2.1	17.5	±	1.5
SKUA-DH-12	114.2	±	4.6	23.5	±	0.8	23.3	±	0.6	80.8	±	2.7	46.0	±	1.3
SKUA-DH-13	97.6	±	3.2	17.6	±	0.1	16.9	±	0.2	114.0	±	4.8	48.0	±	3.5
SKUA-DH-14	102.4	±	4.4	18.0	±	0.3	23.8	±	0.7	64.9	±	3.6	28.8	±	3.3
SKUA-DH-15	111.4	±	2.2	19.1	±	0.3	17.1	±	0.1	78.5	±	3.1	37.1	±	2.6
SKUA-DH-16	82.4	±	3.6	13.4	±	0.4	12.4	±	0.0	52.8	±	2.8	16.9	±	1.7
SKUA-DH-17	94.1	±	2.9	10.8	±	0.6	18.9	±	1.0	89.1	±	3.0	22.3	±	2.7
SKUA-DH-18	83.3	±	2.7	22.6	±	0.3	15.6	±	0.2	56.8	±	3.2	30.8	±	3.5
SKUA-DH-19	94.9	±	4.7	14.6	±	0.0	14.1	±	0.8	100.5	±	3.0	35.0	±	1.2
SKUA-DH-20	85.7	±	3.1	21.8	±	0.6	13.7	±	0.2	64.1	±	4.1	33.6	±	2.3
SKUA-DH-21	82.0	±	5.1	18.4	±	0.9	12.5	±	0.8	108.0	±	3.9	48.2	±	2.5
SKUA-DH-22	80.2	±	2.1	24.3	±	0.3	23.0	±	0.2	62.2	±	3.6	37.2	±	2.6
SKUA-DH-23	98.0	±	3.9	16.1	±	0.7	22.5	±	0.9	83.6	±	4.0	33.2	±	3.5
SKUA-DH-24	97.8	±	2.9	18.7	±	0.6	23.6	±	0.2	62.4	±	3.9	27.9	±	3.3
SKUA-DH-25	87.6	±	5.1	15.2	±	0.4	23.3	±	0.7	98.3	±	3.2	36.8	±	1.8
SKUA-DH-26	98.0	±	3.7	18.5	±	0.0	12.4	±	0.6	94.7	±	4.6	42.3	±	3.6
SKUA-DH-27	104.0	±	2.5	20.1	±	0.3	22.5	±	0.7	114.4	±	4.4	57.0	±	1.3

SKUA-DH-28	114.7	±	3.1	22.8	±	0.6	23.1	±	0.0	80.8	±	4.1	44.0	±	3.7
SKUA-DH-29	82.7	±	5.8	14.5	±	0.8	22.3	±	0.6	71.4	±	3.3	24.9	±	3.3
SKUA-DH-30	94.3	±	3.2	14.2	±	0.5	14.9	±	0.4	65.3	±	4.9	22.8	±	1.2
SKUA-DH-31	76.8	±	5.0	16.8	±	0.8	14.7	±	0.1	66.7	±	2.6	26.4	±	3.0
SKUA-DH-32	87.4	±	2.9	22.2	±	0.5	22.1	±	0.7	77.1	±	2.4	42.4	±	2.2
SKUA-DH-33	107.1	±	3.9	26.0	±	0.7	22.2	±	0.7	105.6	±	2.0	65.6	±	1.6
SKUA-DH-34	85.5	±	4.9	22.9	±	1.0	15.7	±	0.9	72.5	±	4.3	39.6	±	3.7
SKUA-DH-35	113.4	±	2.7	19.9	±	0.4	24.7	±	0.2	55.1	±	3.5	26.1	±	2.5
SKUA-DH-36	107.9	±	3.4	16.0	±	0.5	13.5	±	0.6	102.9	±	2.5	40.8	±	3.6
SKUA-DH-37	80.9	±	2.8	15.8	±	0.2	14.8	±	0.8	76.1	±	3.4	28.5	±	1.7
SKUA-DH-38	80.2	±	2.4	13.2	±	0.2	13.4	±	0.9	90.5	±	3.2	29.3	±	3.5
SKUA-DH-39	113.7	±	3.2	10.8	±	0.1	16.8	±	0.9	82.2	±	4.4	20.5	±	2.4
SKUA-DH-40	103.1	±	4.0	17.2	±	0.4	19.1	±	0.9	56.5	±	4.1	23.8	±	2.9
SKUA-DH-41	97.8	±	2.1	19.1	±	0.4	22.7	±	0.4	66.8	±	2.7	31.4	±	3.8
SKUA-DH-42	113.1	±	4.8	11.3	±	0.9	13.4	±	0.6	100.4	±	3.1	27.5	±	3.3
SKUA-DH-43	111.8	±	3.7	16.7	±	0.6	19.5	±	0.0	65.1	±	3.5	26.0	±	3.1
SKUA-DH-44	92.4	±	2.8	10.4	±	0.4	23.3	±	0.5	79.2	±	3.0	19.8	±	2.8
SKUA-DH-45	113.1	±	3.5	12.6	±	0.0	24.1	±	0.9	76.6	±	2.0	22.8	±	1.7
SKUA-DH-46	94.2	±	4.9	10.8	±	0.7	12.9	±	0.9	87.0	±	3.6	21.8	±	3.5
SKUA-DH-47	81.2	±	4.2	19.3	±	0.8	20.7	±	0.7	59.9	±	3.7	28.0	±	1.5
SKUA-DH-48	109.5	±	5.6	16.0	±	0.4	14.8	±	0.6	111.2	±	3.5	41.6	±	2.8
SKUA-DH-49	102.2	±	5.1	10.2	±	0.5	24.2	±	0.4	110.1	±	4.0	27.5	±	1.5
SKUA-DH-50	83.5	±	5.6	14.4	±	0.5	12.1	±	0.9	62.9	±	4.6	21.7	±	1.7
SKUA-DH-51	103.4	±	4.6	12.6	±	0.2	12.5	±	0.7	105.1	±	2.1	31.5	±	2.6
SKUA-DH-52	114.3	±	3.4	16.4	±	0.2	21.4	±	0.0	56.1	±	3.9	22.4	±	1.5
SKUA-DH-53	112.4	±	2.5	24.4	±	0.7	23.7	±	0.4	84.6	±	2.6	50.4	±	1.9
SKUA-DH-54	108.8	±	2.3	23.5	±	0.6	18.2	±	0.2	91.7	±	3.7	52.3	±	3.7
SKUA-DH-55	75.9	±	3.7	23.6	±	0.5	15.2	±	0.2	109.8	±	2.4	62.7	±	1.1
SKUA-DH-56	102.5	±	3.6	19.2	±	0.7	23.8	±	0.3	111.4	±	3.0	52.7	±	3.7
SKUA-DH-57	82.3	±	3.1	24.3	±	0.6	17.6	±	0.0	55.5	±	3.8	33.0	±	3.7
SKUA-DH-58	114.6	±	2.1	17.7	±	0.3	16.1	±	1.0	84.0	±	2.6	35.7	±	3.7

SKUA-DH-59	74.6	±	3.6	25.4	±	0.4	23.4	±	0.2	90.2	±	3.8	56.3	±	2.3
SKUA-DH-60	106.6	±	4.0	14.8	±	0.2	16.8	±	0.0	102.2	±	2.1	35.7	±	2.5
SKUA-DH-61	79.7	±	5.3	10.5	±	0.7	19.0	±	0.8	59.5	±	4.8	14.8	±	2.2
SKUA-DH-62	104.5	±	4.5	22.7	±	0.7	23.9	±	0.4	100.0	±	4.9	54.5	±	3.8
SKUA-DH-63	81.4	±	3.3	22.0	±	0.1	14.9	±	0.1	89.1	±	2.8	46.7	±	2.0
SKUA-DH-64	105.1	±	3.0	12.8	±	0.3	12.3	±	0.2	85.8	±	3.6	25.5	±	3.4
SKUA-DH-65	80.3	±	5.6	14.8	±	0.2	17.9	±	0.4	61.4	±	2.1	21.4	±	2.5
SKUA-DH-66	107.6	±	3.1	20.5	±	0.2	20.8	±	0.3	87.7	±	4.0	43.5	±	3.6
SKUA-DH-67	80.3	±	2.7	18.2	±	0.6	24.9	±	0.5	58.1	±	4.0	26.1	±	2.2
SKUA-DH-68	82.1	±	2.0	20.4	±	0.3	17.2	±	0.1	76.9	±	2.5	38.0	±	1.2
SKUA-DH-69	76.0	±	5.3	20.0	±	1.0	24.3	±	0.3	85.0	±	2.8	39.9	±	2.6
SKUA-DH-70	94.3	±	3.5	12.6	±	0.8	17.4	±	0.8	114.2	±	3.9	34.2	±	3.2
SKUA-DH-71	106.1	±	3.6	24.6	±	0.8	14.4	±	0.2	77.1	±	4.4	46.2	±	1.9
SKUA-DH-72	83.4	±	2.3	18.9	±	0.8	19.2	±	0.7	87.3	±	3.6	39.2	±	1.4
SKUA-DH-73	97.1	±	2.8	17.2	±	0.6	21.6	±	0.2	93.4	±	3.7	39.5	±	2.4
SKUA-DH-74	85.8	±	2.6	14.3	±	0.6	22.7	±	0.9	104.1	±	2.7	36.4	±	2.7
SKUA-DH-75	80.7	±	5.7	17.0	±	0.4	15.0	±	0.9	110.1	±	4.3	46.8	±	3.0
SKUA-DH-76	115.8	±	2.3	19.4	±	0.0	15.7	±	0.5	67.5	±	2.6	31.8	±	3.6
SKUA-DH-77	78.6	±	3.1	14.4	±	0.3	17.4	±	0.7	58.3	±	4.7	20.3	±	1.2
SKUA-DH-78	105.5	±	2.4	16.9	±	0.4	22.2	±	0.1	76.5	±	3.4	30.4	±	2.9
SKUA-DH-79	80.3	±	5.0	24.9	±	0.9	24.5	±	0.9	102.8	±	3.7	61.2	±	3.2
SKUA-DH-80	89.8	±	2.4	26.0	±	0.0	22.1	±	0.2	89.4	±	2.7	55.6	±	1.3
SKUA-DH-81	111.5	±	3.9	15.1	±	0.4	17.7	±	0.6	76.0	±	2.3	28.1	±	1.8
SKUA-DH-82	79.5	±	4.1	25.1	±	0.9	22.4	±	0.5	103.5	±	3.3	64.4	±	3.8
SKUA-DH-83	113.4	±	5.1	25.7	±	0.2	24.9	±	0.9	84.3	±	4.6	52.5	±	1.9
SKUA-DH-84	89.8	±	5.3	19.8	±	0.1	21.6	±	0.5	97.8	±	4.3	46.1	±	2.8
SKUA-DH-85	82.5	±	4.0	19.0	±	0.2	20.9	±	0.9	59.5	±	3.2	26.6	±	2.9
SKUA-DH-86	98.1	±	5.0	24.2	±	0.8	23.3	±	0.2	62.3	±	2.4	37.2	±	3.4
SKUA-DH-87	107.2	±	2.1	24.6	±	0.3	22.4	±	0.4	59.1	±	2.0	35.4	±	2.7
SKUA-DH-88	84.7	±	2.9	13.4	±	0.5	12.2	±	0.0	101.4	±	3.4	32.8	±	3.2
SKUA-DH-89	94.0	±	3.1	15.8	±	0.4	17.3	±	0.7	95.8	±	3.5	35.6	±	2.9

SKUA-DH-90	102.7 ± 4.8	19.9 ± 0.8	20.0 ± 0.4	68.8 ± 4.3	32.3 ± 1.7
SKUA-DH-91	74.8 ± 2.2	17.5 ± 0.9	21.7 ± 0.9	102.5 ± 3.8	43.4 ± 2.3
SKUA-DH-92	99.7 ± 3.8	24.1 ± 0.8	15.7 ± 0.5	73.4 ± 5.0	43.8 ± 1.0
SKUA-DH-93	78.7 ± 3.5	25.2 ± 1.0	18.9 ± 0.2	70.9 ± 3.9	43.8 ± 1.7
SKUA-DH-94	86.9 ± 2.1	21.6 ± 0.4	13.0 ± 0.8	71.4 ± 2.9	37.3 ± 1.5
SKUA-DH-95	112.2 ± 4.0	19.4 ± 0.2	20.4 ± 0.7	71.1 ± 3.9	33.7 ± 3.9
SKUA-DH-96	109.0 ± 2.8	12.4 ± 1.0	20.2 ± 0.8	63.9 ± 3.8	18.9 ± 3.3
SKUA-DH-97	74.4 ± 5.5	10.9 ± 0.4	17.3 ± 0.2	91.0 ± 2.9	22.8 ± 1.6
SKUA-DH-98	85.8 ± 3.1	15.3 ± 0.5	21.9 ± 0.2	111.7 ± 2.2	41.6 ± 3.4
SKUA-DH-99	77.8 ± 4.2	21.7 ± 0.5	23.6 ± 0.5	90.6 ± 2.3	47.3 ± 2.9
SKUA-DH-100	111.6 ± 3.2	10.9 ± 0.4	23.3 ± 0.7	103.5 ± 4.8	25.8 ± 1.5
SKUA-DH-101	86.8 ± 3.9	12.6 ± 0.3	25.0 ± 0.0	52.6 ± 3.6	15.6 ± 3.0
SKUA-DH-102	111.0 ± 4.3	22.7 ± 0.6	20.9 ± 0.8	72.2 ± 3.0	39.6 ± 1.8
SKUA-DH-103	99.6 ± 2.9	15.6 ± 0.8	23.1 ± 0.2	89.7 ± 3.1	33.4 ± 1.8
SKUA-DH-104	97.8 ± 4.9	25.2 ± 0.9	14.1 ± 0.4	79.2 ± 3.5	49.4 ± 3.6
SKUA-DH-105	97.9 ± 3.1	13.3 ± 1.0	24.7 ± 0.3	95.2 ± 2.5	30.9 ± 3.3
SKUA-DH-106	109.4 ± 4.8	21.9 ± 0.8	13.9 ± 0.3	72.5 ± 3.2	37.8 ± 2.9
SKUA-DH-107	80.3 ± 2.5	19.5 ± 0.4	15.7 ± 0.3	108.9 ± 3.2	51.3 ± 2.1
SKUA-DH-108	90.1 ± 5.5	12.3 ± 0.2	16.2 ± 0.9	69.8 ± 3.8	20.7 ± 3.0
SKUA-DH-109	80.3 ± 4.1	11.5 ± 0.3	20.2 ± 0.3	82.7 ± 4.6	22.6 ± 3.7
SKUA-DH-110	104.1 ± 3.0	18.6 ± 0.8	14.7 ± 0.5	108.5 ± 4.3	48.6 ± 3.1
SKUA-DH-111	94.9 ± 3.8	21.2 ± 0.6	17.7 ± 0.8	94.3 ± 4.0	49.4 ± 2.1
SKUA-DH-112	92.1 ± 4.1	17.1 ± 0.5	22.5 ± 0.2	56.1 ± 2.9	23.8 ± 3.7
SKUA-DH-113	111.0 ± 5.5	24.7 ± 0.1	14.0 ± 0.1	92.0 ± 4.1	54.6 ± 1.7
SKUA-DH-114	115.7 ± 4.4	17.7 ± 0.4	12.2 ± 0.2	59.0 ± 2.5	25.1 ± 4.0
SKUA-DH-115	75.9 ± 2.0	16.2 ± 0.2	12.6 ± 0.5	57.2 ± 3.4	22.8 ± 2.7
SKUA-DH-116	78.9 ± 2.3	18.9 ± 0.3	13.2 ± 0.7	107.2 ± 2.4	48.2 ± 2.8
SKUA-DH-117	74.3 ± 3.8	14.7 ± 0.2	13.7 ± 0.2	56.7 ± 4.6	19.6 ± 1.0
SKUA-DH-118	78.3 ± 5.1	23.2 ± 0.1	14.3 ± 0.5	59.8 ± 4.1	33.9 ± 2.3
SKUA-DH-119	89.4 ± 3.6	11.1 ± 0.9	13.9 ± 0.5	60.1 ± 3.8	16.5 ± 1.4
SKUA-DH-120	99.9 ± 3.5	25.2 ± 0.1	19.7 ± 0.6	77.9 ± 3.1	48.1 ± 1.3

SKUA-DH-121	96.1	±	4.8	21.2	±	0.2	20.8	±	0.3	78.1	±	4.5	41.0	±	1.1
SKUA-DH-122	97.7	±	4.5	22.0	±	0.9	14.8	±	0.3	105.1	±	4.9	57.8	±	1.5
SKUA-DH-123	80.0	±	5.2	10.6	±	0.2	23.8	±	1.0	77.1	±	4.0	19.3	±	2.6
SKUA-DH-124	114.6	±	4.6	10.5	±	1.0	24.2	±	0.5	103.4	±	4.0	25.8	±	1.4
SKUA-DH-125	99.4	±	4.1	13.5	±	0.8	15.6	±	0.2	62.9	±	2.2	20.2	±	1.9
SKUA-DH-126	91.0	±	3.5	14.3	±	0.1	20.2	±	0.9	110.3	±	4.2	38.5	±	3.8
SKUA-DH-127	79.6	±	2.5	19.4	±	0.4	22.8	±	0.9	108.5	±	3.1	51.3	±	2.6
SKUA-DH-128	112.2	±	3.7	16.3	±	0.1	16.9	±	1.0	107.6	±	3.1	42.8	±	3.2
SKUA-DH-129	77.5	±	4.5	15.8	±	0.9	18.9	±	0.0	75.9	±	3.7	28.1	±	3.2
SKUA-DH-130	90.8	±	6.0	16.1	±	0.3	18.8	±	0.6	106.9	±	4.6	42.4	±	2.9
SKUA-DH-131	115.7	±	3.3	15.6	±	0.9	22.6	±	0.4	112.5	±	2.8	42.0	±	1.2
SKUA-DH-132	77.9	±	2.4	11.4	±	0.1	18.7	±	0.5	102.4	±	2.3	28.1	±	3.3
SKUA-DH-133	77.1	±	4.7	11.7	±	0.5	14.4	±	1.0	109.1	±	3.0	30.0	±	2.7
SKUA-DH-134	93.1	±	3.1	16.0	±	1.0	23.4	±	0.7	92.3	±	2.2	36.8	±	2.9
SKUA-DH-135	95.4	±	4.4	25.8	±	0.7	13.8	±	0.8	82.6	±	2.2	51.3	±	3.2
SKUA-DH-136	90.0	±	3.2	23.7	±	0.9	20.2	±	0.1	74.1	±	3.7	42.6	±	1.6
SKUA-DH-137	100.7	±	3.8	13.9	±	0.4	17.6	±	0.2	69.3	±	3.5	22.4	±	3.7
SKUA-DH-138	76.0	±	5.8	15.1	±	0.5	15.2	±	1.0	80.5	±	2.5	30.0	±	1.3
SKUA-DH-139	95.3	±	4.6	15.7	±	0.7	13.8	±	0.9	86.1	±	2.5	32.3	±	1.8
SKUA-DH-140	85.8	±	4.6	17.7	±	0.0	24.3	±	0.3	115.7	±	3.0	48.9	±	1.2
SKUA-DH-141	111.3	±	4.3	11.7	±	0.1	18.2	±	0.4	100.1	±	4.6	27.5	±	3.4
SKUA-DH-142	89.4	±	4.2	22.2	±	0.5	12.1	±	0.3	59.8	±	3.7	32.5	±	2.6
SKUA-DH-143	99.9	±	3.2	25.5	±	0.8	14.1	±	0.7	92.3	±	3.5	57.5	±	2.2
SKUA-DH-144	92.4	±	5.4	21.5	±	0.5	12.1	±	0.0	114.0	±	4.8	59.3	±	2.6
SKUA-DH-145	76.3	±	5.2	23.1	±	0.8	13.9	±	0.7	95.6	±	4.0	54.6	±	2.6
SKUA-DH-146	77.0	±	2.1	19.6	±	0.3	18.5	±	0.4	112.6	±	4.4	53.2	±	3.8
SKUA-DH-147	84.1	±	4.3	14.9	±	0.3	17.3	±	0.2	55.9	±	3.4	19.3	±	3.5
SKUA-DH-148	115.0	±	3.7	11.1	±	0.2	23.9	±	0.9	116.0	±	4.8	31.6	±	1.6
SKUA-DH-149	107.6	±	3.8	11.4	±	0.9	20.3	±	0.1	106.1	±	2.3	29.2	±	3.4
SKUA-DH-150	93.7	±	5.1	18.8	±	0.2	22.1	±	0.7	53.4	±	4.1	23.9	±	1.9
SKUA-DH-151	114.2	±	3.6	18.9	±	0.7	24.4	±	0.9	100.1	±	2.4	45.0	±	1.2

SKUA-DH-152	92.1	±	2.2	22.7	±	0.7	19.5	±	0.4	100.9	±	2.5	55.0	±	3.1
SKUA-DH-153	113.8	±	3.5	19.2	±	0.5	17.1	±	0.0	54.3	±	4.1	25.7	±	3.5
SKUA-DH-154	86.1	±	5.6	17.1	±	0.2	14.7	±	0.4	102.8	±	4.6	43.4	±	1.4
SKUA-DH-155	92.6	±	5.1	17.7	±	1.0	21.8	±	0.4	61.5	±	4.6	25.9	±	3.7
SKUA-DH-156	101.8	±	3.1	12.2	±	0.5	18.7	±	0.7	95.2	±	2.2	28.5	±	1.6
SKUA-DH-157	103.0	±	3.1	24.8	±	0.3	22.8	±	0.6	85.8	±	4.6	51.0	±	2.6
SKUA-DH-158	95.3	±	4.0	19.1	±	0.8	24.9	±	0.2	78.7	±	3.1	37.1	±	1.3
SKUA-DH-159	109.1	±	3.5	14.1	±	0.8	22.3	±	0.2	88.7	±	3.2	30.8	±	1.3
SKUA-DH-160	91.2	±	4.5	12.0	±	0.4	12.7	±	0.6	56.0	±	3.7	15.4	±	2.5
SKUA-DH-161	115.5	±	4.8	15.7	±	0.6	22.2	±	0.7	77.7	±	4.1	28.9	±	1.9
SKUA-DH-162	96.8	±	2.9	25.4	±	0.4	18.6	±	0.3	75.1	±	4.2	46.9	±	3.9
SKUA-DH-163	109.9	±	2.7	13.2	±	0.3	21.7	±	0.5	85.3	±	2.6	27.6	±	1.9
SKUA-DH-164	92.6	±	2.6	10.1	±	0.1	23.7	±	0.3	114.6	±	2.1	28.5	±	1.4
SKUA-DH-165	79.3	±	3.9	11.3	±	0.6	21.9	±	0.2	88.4	±	3.9	24.2	±	3.3
SKUA-DH-166	97.3	±	5.5	23.6	±	0.3	14.7	±	0.9	53.4	±	2.3	30.5	±	3.2
SKUA-DH-167	91.7	±	3.4	21.1	±	1.0	21.2	±	0.8	98.6	±	2.5	51.5	±	3.4
SKUA-DH-168	92.9	±	3.0	10.9	±	0.9	24.4	±	0.0	71.3	±	2.6	17.8	±	2.5
SKUA-DH-169	111.6	±	5.0	15.4	±	0.6	20.3	±	0.3	90.1	±	2.8	33.8	±	1.8
SKUA-DH-170	93.0	±	2.7	24.2	±	0.1	12.3	±	0.5	68.6	±	4.7	40.8	±	3.3
SKUA-DH-171	74.3	±	3.6	18.5	±	0.1	21.9	±	0.4	114.3	±	4.9	51.3	±	2.5
SKUA-DH-172	96.0	±	2.8	12.2	±	0.7	19.6	±	0.7	62.6	±	3.9	18.6	±	3.5
SKUA-DH-173	87.2	±	2.7	14.2	±	0.1	24.2	±	0.8	99.1	±	3.5	34.7	±	3.1
SKUA-DH-174	112.5	±	3.8	14.8	±	1.0	12.9	±	0.5	94.4	±	4.1	32.9	±	3.6
SKUA-DH-175	94.3	±	2.2	19.7	±	0.9	15.3	±	0.1	115.5	±	3.3	54.6	±	1.7
SKUA-DH-176	76.5	±	5.2	25.9	±	0.7	21.8	±	0.2	89.3	±	3.0	55.6	±	2.4
SKUA-DH-177	110.0	±	4.9	12.2	±	0.2	12.7	±	0.7	83.7	±	3.1	24.9	±	2.8
SKUA-DH-178	109.9	±	2.9	23.1	±	0.2	15.8	±	0.8	107.0	±	3.1	61.5	±	2.6
SKUA-DH-179	104.2	±	3.4	10.8	±	0.6	23.9	±	0.3	74.0	±	3.2	18.3	±	1.4
SKUA-DH-180	84.2	±	2.7	21.7	±	0.1	13.2	±	0.1	70.4	±	3.2	36.8	±	2.7
SKUA-DH-181	105.8	±	5.4	17.6	±	0.2	13.0	±	0.3	114.7	±	2.4	48.5	±	3.1
SKUA-DH-182	103.9	±	4.6	24.1	±	0.8	19.1	±	0.3	84.4	±	4.3	50.4	±	2.7

SKUA-DH-183	111.9	±	2.7	13.8	±	0.3	21.5	±	0.2	54.0	±	5.0	17.6	±	1.1
SKUA-DH-184	108.6	±	3.8	17.3	±	0.7	24.3	±	0.0	68.5	±	3.7	28.9	±	1.9
SKUA-DH-185	83.2	±	5.8	20.1	±	0.3	13.7	±	0.8	86.6	±	4.0	43.0	±	4.0
SKUA-DH-186	104.9	±	2.7	12.3	±	0.2	20.3	±	0.2	87.1	±	2.8	26.1	±	3.8
SKUA-DH-187	107.5	±	3.6	10.3	±	0.9	18.8	±	0.4	55.9	±	2.7	13.8	±	2.7
SKUA-DH-188	89.8	±	6.0	20.9	±	0.2	19.1	±	0.9	55.8	±	2.7	27.5	±	2.4
SKUA-DH-189	77.7	±	5.9	16.6	±	0.0	12.0	±	0.3	97.8	±	3.2	38.8	±	2.3
SKUA-DH-190	79.5	±	2.7	17.9	±	0.9	14.2	±	0.6	98.7	±	2.7	41.7	±	1.3
SKUA-DH-191	90.3	±	2.8	24.5	±	0.2	12.1	±	0.8	115.1	±	2.9	69.0	±	1.8
SKUA-DH-192	74.6	±	5.8	18.8	±	0.7	22.5	±	0.4	60.1	±	4.6	27.0	±	2.4
SKUA-DH-193	75.1	±	5.4	23.9	±	0.8	19.7	±	0.8	103.3	±	4.4	59.2	±	3.8
SKUA-DH-194	74.1	±	4.4	21.4	±	0.8	18.4	±	0.1	63.9	±	3.4	33.1	±	3.8
SKUA-DH-195	74.5	±	3.1	17.4	±	0.7	24.7	±	0.7	66.1	±	3.3	28.1	±	1.4
SKUA-DH-196	79.9	±	5.6	25.4	±	0.4	14.7	±	0.7	58.5	±	4.1	36.3	±	1.9
SKUA-DH-197	93.7	±	4.6	25.7	±	0.5	15.9	±	0.9	78.6	±	3.1	48.8	±	2.7
SKUA-DH-198	88.6	±	2.4	10.3	±	0.7	24.7	±	0.2	101.7	±	3.5	25.3	±	2.8
SKUA-DH-199	108.1	±	4.3	14.6	±	0.3	13.6	±	0.6	58.5	±	2.4	20.3	±	2.0
SKUA-DH-200	91.8	±	5.0	14.1	±	0.6	23.1	±	0.8	70.3	±	4.1	24.5	±	1.7
SKUA-DH-201	85.2	±	3.5	15.9	±	0.3	20.0	±	0.7	81.7	±	2.6	30.4	±	2.7
SKUA-DH-202	76.4	±	4.8	11.6	±	0.1	20.5	±	0.3	54.7	±	4.1	14.9	±	3.3
SKUA-DH-203	101.3	±	3.4	22.1	±	0.1	18.5	±	0.3	90.7	±	4.3	49.5	±	1.8
SKUA-DH-204	91.0	±	4.6	22.0	±	0.6	14.5	±	1.0	84.4	±	3.2	44.1	±	3.3
SKUA-DH-205	106.9	±	4.7	20.0	±	0.4	22.9	±	0.6	83.2	±	3.5	41.5	±	2.9
SKUA-DH-206	74.4	±	5.6	10.3	±	0.6	21.5	±	0.4	82.8	±	2.9	20.5	±	3.4
SKUA-DH-207	94.9	±	3.8	12.9	±	0.6	19.3	±	0.8	52.9	±	3.6	15.6	±	3.9
**Mean**	**94.6**	**±**	**3.8**	**17.8**	**±**	**0.5**	**18.8**	**±**	**0.5**	**83.9**	**±**	**3.5**	**36.2**	**±**	**2.5**
**Max**	**115.8**			**26.0**			**25.0**			**116.0**			**69.0**		
**Min**	**74.1**			**10.1**			**12.0**			**52.6**			**13.3**		
**K-332 (Female Parent)**	**91.1**	**±**	**2.4**	**15.7**	**±**	**0.5**	**15.2**	**±**	**0.7**	**104.5**	**±**	**4.6**	**45.6**	**±**	**1.1**
**GS-88 Male Parent)**	**113.2**	**±**	**3.2**	**12.3**	**±**	**0.2**	**18.3**	**±**	**0.5**	**98.3**	**±**	**3.9**	**23.7**	**±**	**1.9**
