# Supplementary material for: Improved protocol for efficacious in vitro androgenesis and development of doubled haploids in temperate japonica rice
Source: PLoS One. 2020 Nov 2;15(11):e0241292. doi: 10.1371/journal.pone.0241292 (PMC7605686; doi:10.1371/journal.pone.0241292)
Supplement: S1 Raw images — (PDF) [file pone.0241292.s009.pdf]

Fig 1. Confirmation of hybridity of F_1 individuals with polymorphic SSR marker RM72.

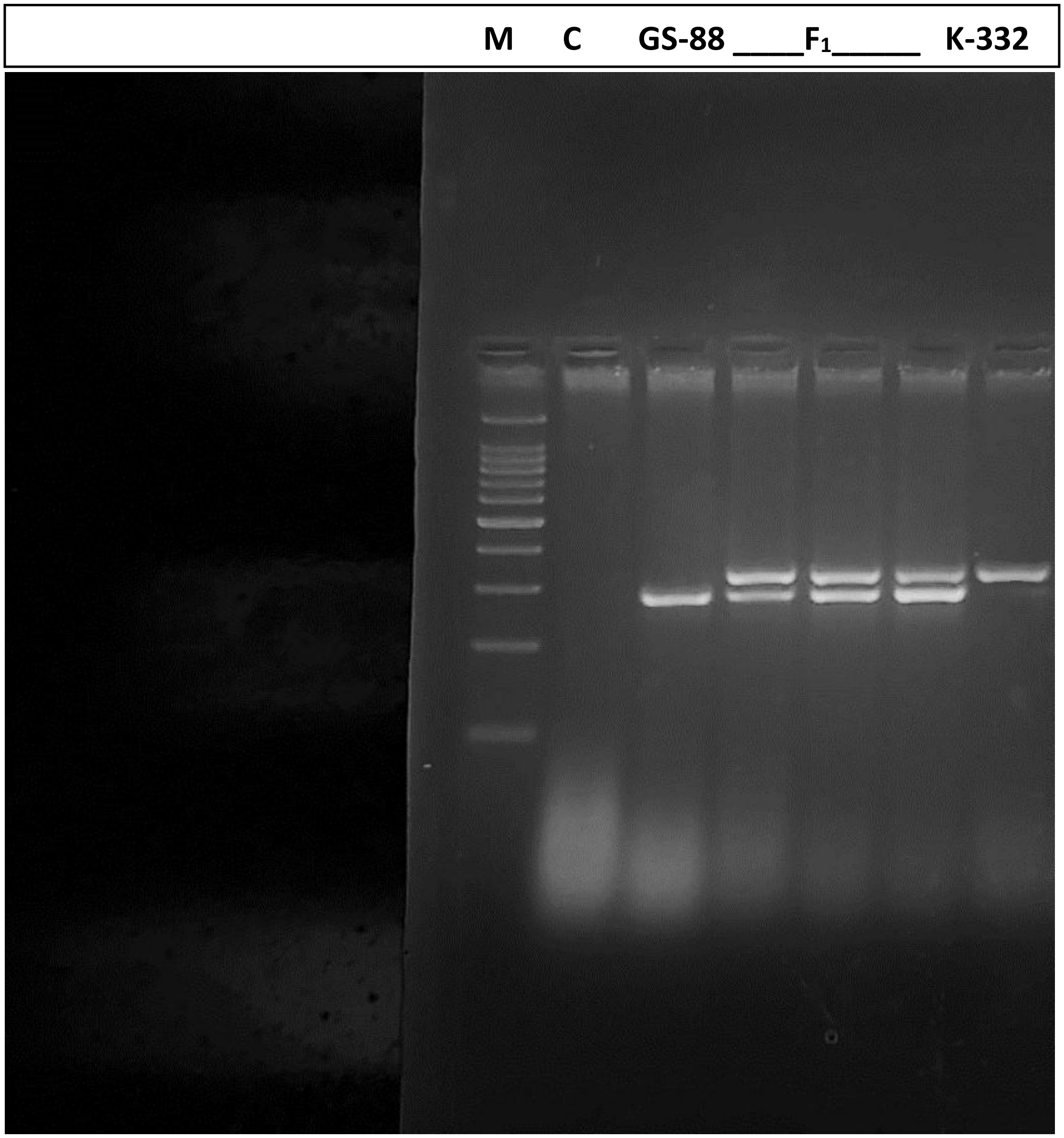

M: 100 bp DNA Ladder (Genetix Biotech Asia, New Delhi, India), C: blank

S3 Fig. Polymorphism survey between GS-88 and K-332 using SSR markers distributed on different chromosomes

M	RM132	RM6100	RM19	RM169	RM1600	RM72	RM1337	X	X	X

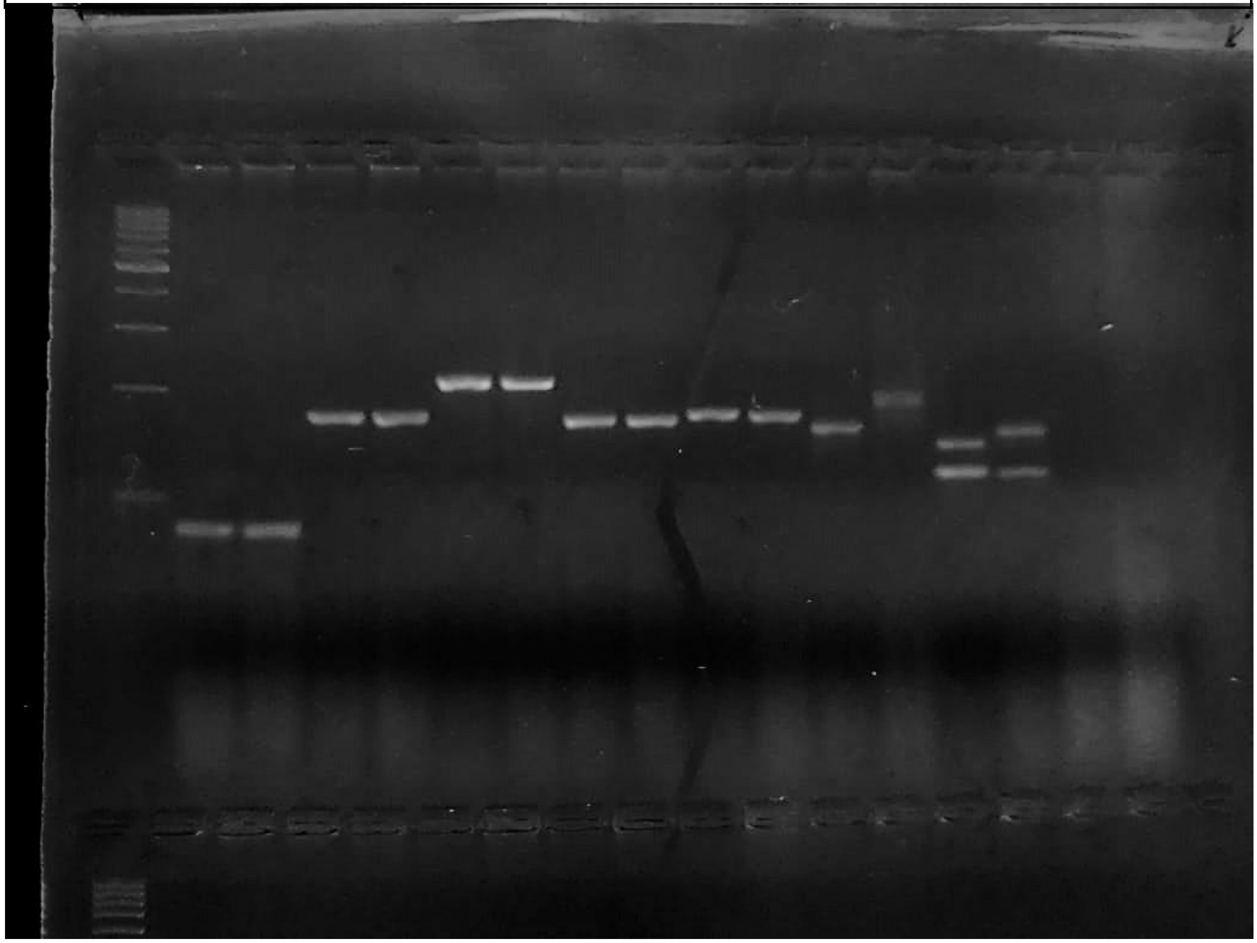

M: 100 bp DNA Ladder (Thermo Fisher Scientific, New Delhi, India); Left well: GS-88; Right well: K-332
